# Recent Progress in NIR-II Contrast Agent for Biological Imaging

**DOI:** 10.3389/fbioe.2019.00487

**Published:** 2020-01-30

**Authors:** Jie Cao, Binling Zhu, Kefang Zheng, Songguo He, Liang Meng, Jibin Song, Huanghao Yang

**Affiliations:** ^1^Fuzhou University Postdoctoral Research Station of Chemical Engineering and Technology, Fuzhou University, Fuzhou, China; ^2^Scientific Research and Experiment Center, Fujian Police College, Fuzhou, China; ^3^Fujian Police College Judicial Expertise Center, Fuzhou, China; ^4^Department of Forensic Science, Fujian Police College, Fuzhou, China; ^5^Engineering Research Center, Fujian Police College, Fuzhou, China; ^6^The Key Lab of Analysis and Detection Technology for Food Safety of the MOE and Fujian Province, State Key Laboratory of Photocatalysis on Energy and Environment, College of Chemistry, Fuzhou University, Fuzhou, China

**Keywords:** fluorescence imaging technology, the second region near infrared (NIR-II), biological imaging, contrast agents, biomedical applications

## Abstract

Fluorescence imaging technology has gradually become a new and promising tool for *in vivo* visualization detection. Because it can provide real-time sub-cellular resolution imaging results, it can be widely used in the field of biological detection and medical detection and treatment. However, due to the limited imaging depth (1–2 mm) and self-fluorescence background of tissue emitted in the visible region (400–700 nm), it fails to reveal biological complexity in deep tissues. The traditional near infrared wavelength (NIR-I, 650–950 nm) is considered as the first biological window, because it reduces the NIR absorption and scattering from blood and water in organisms. NIR fluorescence bioimaging's penetration is larger than that of visible light. In fact, NIR-I fluorescence bioimaging is still interfered by tissue autofluorescence (background noise), and the existence of photon scattering, which limits the depth of tissue penetration. Recent experimental and simulation results show that the signal-to-noise ratio (SNR) of bioimaging can be significantly improved at the second region near infrared (NIR-II, 1,000–1,700 nm), also known as the second biological window. NIR-II bioimaging is able to explore deep-tissues information in the range of centimeter, and to obtain micron-level resolution at the millimeter depth, which surpass the performance of NIR-I fluorescence imaging. The key of fluorescence bioimaging is to achieve highly selective imaging thanks to the functional/targeting contrast agent (probe). However, the progress of NIR-II probes is very limited. To date, there are a few reports about NIR-II fluorescence probes, such as carbon nanotubes, Ag_2_S quantum dots, and organic small molecular dyes. In this paper, we surveyed the development of NIR-II imaging contrast agents and their application in cancer imaging, medical detection, vascular bioimaging, and cancer diagnosis. In addition, the hotspots and challenges of NIR-II bioimaging are discussed. It is expected that our findings will lay a foundation for further theoretical research and practical application of NIR-II bioimaging, as well as the inspiration of new ideas in this field.

## Introduction

Optical imaging technology has become a very important research method in the field of biomedicine, especially for its ability to monitor biomolecules, cells, tissues, and living organisms in real-time and multi-dimensional visualization. Optical imaging technology has many advantages, in that it is non-invasive and safe, has visualization capabilities and high spatial resolution, has high rapid output, and is a low-cost method. It has been widely used in biomolecular detection imaging, drug distribution metabolic tracking, disease detection and diagnosis (Sevick-Muraca et al., 2002; Hilderbrand and Weissleder, 2010; Chen et al., 2014). Especially in early cancer diagnosis and imaging-guided treatment, optical imaging technology have a good application prospect.

In the process of optical *in vivo* bioimaging, the penetration depth of photons primarily depends on the absorption and scattering of tissue elements. Meanwhile, the fluorescence and scattering photons generated by tissue itself will cause interference noise and background radiation to the photon penetration process. Therefore, fluorescence imaging technology also has limitation in its practical applications in that some active components in organisms (such as melanin, hemoglobin, cytochrome, etc.) have higher light absorption and light scattering within the visible band (400–700 nm), which will reduce the penetration depth of visible light. Because organisms are rich in many luminescent macromolecules (usually located in the visible region), these biomolecules can also produce non-specific fluorescence emissions under visible light excitation, thus interfering with imaging results (Weissleder, 2001).

**Figure 1:**
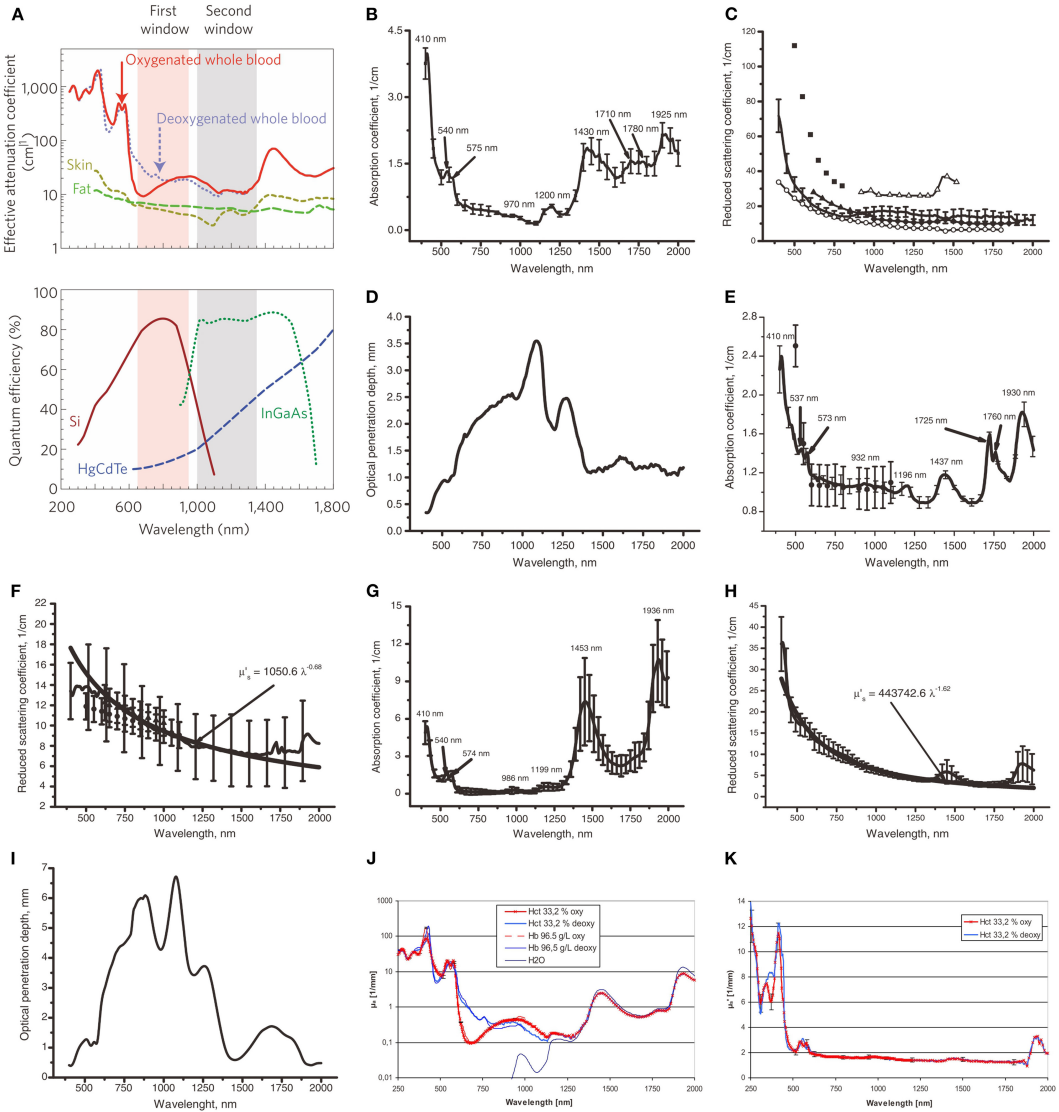
Prahl et al. (1993) reported the inverse double increase (IAD) method and the power law approximation are used to process experimental data and determine the optical properties of tissues. In this figure, μ'_s_ is calculated as μ'_s_ = μ_s_ (1-g), where μ_s_ is the scattering coefficient and g is the anisotropic coefficient of scattering. The solid line corresponds to the average experimental data, and the vertical line shows the SD value. (**A** Top) NIR-I (first window) and NIR-II (second window) imaging windows (Smith et al., 2009). The effective attenuation coefficient represents the how easily a volume of material can be penetrated by a beam of light. (**A** Bottom) The sensitivity curves of the sensor in the signal detector camera with silicon (Si), indium gallium arsenic (InGaAs), and mercury telluride cadmium (HgCdTe). Unlike the charge coupled device (CCD) camera using silicon sensor, the core component of near infrared camera is semiconductor alloy sensor, including InGaAs and HgCdTe, which has a narrower band gap. In particular, InGaAs cameras exhibit high quantum efficiency when used in the NIR-II window, i.e., high sensitivity. Adapted from Smith et al. (2009) written by Smith, A.M., etc. with permission. **(B,C)** Show the relationship between the incident light wavelength and absorption coefficient (μ_a_) or the reduced light scattering coefficient (μ'_s_) in human skin *in vitro*, respectively. **(B)** Adapted from Bashkatov et al. (2005) with permission. In **(C)**, except for the solid line, the remaining data marker points correspond to the experimental data obtained in reference (Chan et al., 1996; Simpson et al., 1998; Du et al., 2001; Troy and Thennadil, 2001; Bashkatov et al., 2005). Adapted from Chan et al. (1996), Simpson et al. (1998), Du et al. (2001), Troy and Thennadil (2001), and Bashkatov et al. (2005) with permission. **(D,I)** Show the penetration depth (δ) of light to skin and human mucosal tissue in the range of incident light wavelength from 400 to 2,000 nm, respectively. Adapted from Bashkatov et al. (2005) with permission. **(E,F)** Show the relationship between wavelength and μ_a_ or μ'_s_ in subcutaneous adipose tissue, respectively. In **(E)**, all the data markers except the solid line correspond to the experimental data obtained in Peters et al. (1990). Adapted from Peters et al. (1990) with permission. In **(F)**, all the data markers except the solid line correspond to the experimental data obtained in Peters et al. (1990) and Simpson et al. (1998). Adapted from Peters et al. (1990) and Simpson et al. (1998). with permission. **(G,H)** Show the relationship between wavelength and μ_a_ or μ'_s_ in human mucosa, respectively. Adapted from Bashkatov et al. (2005) with permission. **(J)** Shows the relationship between wavelength and μ_a_ for red blood cells (RBC) with or without saturated oxygen in the 33.2% hematocrit (HCT) brine solution, as well as the relationship between wavelength and μ_a_ for hemoglobin with or without saturated oxygen in the 96.5 g/dl hemoglobin solution. Adapted from Friebel et al. (2009) with permission. **(J)** Shows the relationship between wavelength and μ'_s_ for RBC with or without saturated oxygen in the 33.2% HCT brine solution. Adapted from Friebel et al. (2009) with permission.

Because near-infrared light (NIR) is less absorbed and scattered in biological tissues than visible light, the former can penetrate biological tissues, such as skin, more effectively. As shown in Figure 1A (top), the effective attenuation coefficients (EACs) of tissue components, such as whole blood (oxygenated or deoxygenated), skin, and fat are significantly lower in the range of 650–950 nm than that in visible light range, which is considered as the “first (optical) window” (NIR-I) (Frangioni, 2003; Smith et al., 2009). In the practice of NIR-I biological imaging, the imaging quality in deep tissue is far from adequate resolution. Due to the large amount of background noise generated by tissue's auto-fluorescence, the tissue penetration depth of photons in NIR-I is only 1–2 cm (Croce and Bottiroli, 2014).

Typically, the NIR-II window that may be suitable for bioimaging application is from 1,000 to 1,700 nm. Tissue components show a strong EAC from 1,350 to 1,700 nm, as shown in Figure 1A (top). We herein narrowed down the wavelength range to 1,000–1,350 nm, as labeled as “second window” in Figure 1A (top). In this review, the “NIR-II” in this manuscript only refers to the wavelength range of 1,000–1,350 nm. The relationship between wavelength and the absorption coefficient or the light scattering coefficient in the skin is shown in Figures 1B,C respectively. The absorption to the skin in NIR-I decreases slowly with the increase of wavelength, and the trend continues into NIR-II. It reaches the lowest point at ~1,125 nm and starts spiking after 1,250 nm. No significant difference is found for the light scattering coefficients in NIR-I and NIR-II regions. The penetration depth of light to the skin can be seen from Figure 1D, from which we could find a sweet spot (largest penetration depth) of 1,125 nm in NIR-II. Overall, the range of 1,000–1,125 nm in NIR-II shows a better performance than NIR-I for skin. We may get the similar conclusions for human subcutaneous fat tissue (Figures 1E,F) and human subcutaneous adipose tissue (Figures 1G–I). Again, it is difficult to compare directly the performance of NIR-I and NIR-II because the absorption coefficients in the latter shows a turning point in the middle of NIR-II (at ~1,125 nm). The sweet spot of 1,000–1,125 nm of NIR-II shows the lowest absorption coefficients for both red blood cells (RBCs) and hemoglobin with or without saturated oxygen, which is also applicable for water (see Figure 1J). In fact, it can be seen from Figure 1J of the relationship between water absorption coefficient with wavelength that the wavelength below 800 nm is the least absorbed by water, which is the optimal excitation wavelength. The closest point in NIR-II to eliminate the absorption from water is at 1,100 nm. It also can be seen from Figure 1K that the effective light scattering of 100% and 0% oxygen saturation RBCs shows no significant differences in NIR-I and NIR-II. However, the autofluorescence from tissue or endogenous compounds limits the signal-to-noise ratio (SNR) of NIR-I fluorescence imaging. The SNR of NIR-II fluorescence imaging, on the other hand, is enhanced due to the elimination of autofluorescence noises. For example, Lim et al. (2003) studied the optical imaging process in different biological media (including tissue and blood) and simulated the process and established relevant models. From the results of this study, it can be concluded that the SNR of quantum dot fluorescent clusters emitting at 1,320 nm is more than 100 times higher than that of quantum dots emitting at 850 nm. This result aroused great interest of the researchers in exploring biocompatible NIR-II fluorescence probes. Therefore, compared with visible light (400–700 nm) and traditional NIR-I window, NIR-II can avoid background interference such as spontaneous fluorescence and photon scattering (Smith et al., 2009; Welsher et al., 2011). In 2009, Welsher et al. (2009) first reported on a class of single-walled carbon nanotubes (SWCNTs), whose fluorescence emission wavelength is 950–1,600 nm. Further experiments show that such material can be used for imaging in living mice. The fluorescence signal of SWCNTs at 950–1,400 nm can be detected to obtain high-resolution imaging of blood vessels under deep skin tissue. Since then, more and more NIR-II fluorescent probes have been successfully developed and have been applied to biomedical imaging field.

At present, the application of NIR-II imaging window is still not fully utilized, due to the lack of research on probe materials and the restriction of imaging equipment. The Figure 1A (bottom) shows the relationship between incident light wavelength and quantum efficiency when cameras with different sensors are used, including silicon (Si), InGaAs, and HgCdTe. Furthermore, Si camera is the most sensitive to the light of NIR-I, while InGaAs camera is the most sensitive to the light of NIR-II. In addition, HgCdTe camera is less sensitive to the light of these two windows although it has a higher resolution array, and it is most sensitive to the light of longer wavelength. Silicon-based CCD (Si-CCD) is usually used to collect fluorescence signals from NIR-I window. However, the quantum yield of this kind of CCD in NIR-II window is low, which is not sufficient for signal acquisition. Near infrared CCD based on InGaAs is commonly used as a NIR-II window detector, but the high cost of use restricts its widespread use in research (Smith et al., 2009).

In recent years, there have been a few successful applications of Nanofluorescent probes developed by NIR-II technology in bioimaging, which include the use of carbon nanotubes (Hong et al., 2014; Diao et al., 2015), Ag_2_S quantum dots (Hong et al., 2012b; Zhang et al., 2012) and small organic molecules (Antaris et al., 2016). Next, the applications of various near infrared nanomaterials and bioimaging will be introduced in detail.

## Categories of Reported NIR-II Nanomaterials

### Single-Walled Carbon Nanotubes

Semiconductor-based single-walled carbon nanotubes (SWCNTs) have unique optical properties due to the existence of the bandgap. When photons interact with SWCNTs, they are absorbed and released as fluorescence. It is worth noting that the absorption wavelength of SWCNTs is generally in the visible region (400–700 nm) and NIR-I (650–950 nm). However, their emission wavelength is in NIR-II (1,000–1,700 nm), and the energy from absorbed photons is not released by radiation relaxation in the form of heat (O'Connell et al., 2002). SWCNTs has been widely used in the fields of photothermal and photodynamic therapy (Robinson et al., 2010; Murakami et al., 2012), fluorescence labeling, and fluorescence imaging of deep tissue *in vivo* (Welsher et al., 2011; Hong et al., 2012a, 2014). In addition, the use of SWCNT surface functionalization (Zheng et al., 2003) using coating surfactants, polymers, DNA, proteins, and even viruses can greatly enrich the scope of application for SWCNTs in the biological field. However, the characteristically low fluorescence quantum efficiency (<1%) of SWCNTs is a major issue still needing to be addressed.

SWCNTs are used *in vivo* imaging because of their inherently wide NIR-II fluorescence emissions (Yi et al., 2012; Ghosh et al., 2014). In 2014, Belcher's research team coated SWCNTs with an M13 virus, using the M13 virus surface polypeptide, which targets tumor cells, to specifically bind SWCNTs to tumor tissue. Using SWCNTs, Blecher was able to achieve targeted fluorescence imaging of tumor tissues in mice, which proved that the system could be applied to clinical diagnosis (Ghosh et al., 2014). In the same year, Hong et al. combined an IRDye-800 fluorescent group with SWCNTs and intravenously injected the IRDye-800 fluorescent group into mice. Using a NIR-II fluorescence imaging *in vivo* (Hong et al., 2014), they found that the fluorescence of carbon nanotubes could penetrate 2.6 mm deep into a mouse skull, which not only provided imaging of the distribution of blood vessels in the head of mice, but also clearly showed the fine structure of the brain capillaries. In 2019, Toshiya Okazaki and other Japanese scholars used oxygen-doped SWCNTS and enveloped it with phospholipid polyethylene glycol (o-SWCNT-PEG). They found that it has special potential and can emit 1,300 nm NIR-II fluorescence under 980 nm light excitation. Therefore, it is considered to be a promising angiographic imaging probe (Takeuchi et al., 2019). In addition, o-SWCNTs were injected intravenously into living mice as a contrast agent for vascular imaging. The biological factors of the angiographic probe *in vivo*, including retention time, biological distribution, and toxicity, were studied. The results showed that o-SWCNT-PEG, an angiographic probe, had low toxicity *in vivo*, and that it can be successfully used in angiographic imaging within the NIR-II wavelength range.

### Semiconductor Quantum Dots

A second successful application of Nanofluorescent probes developed by NIR-II technology is the use of semiconductor quantum dots. Among the many kinds of semiconductor fluorescent quantum dots available, Ag_2_S quantum dots have been most widely used in near infrared imaging because of their strong NIR-II fluorescence and low biological toxicity. Their biological toxicity is lower than other quantum dots containing Se, Te, Cd, Pb, As, and other acute or chronic toxic elements, and the fluorescence can be tuned from 687 nm in NIR-I to 1,294 nm in NIR-II (Yang et al., 2013; Gui et al., 2014a,b; Zhao and Song, 2014). At the same time, it also has high fluorescence stability and fluorescence quantum efficiency. Ag_2_S quantum dots have smaller particle sizes [3.7 nm (Zhao and Song, 2014), 1.6–6.8 nm (Yang et al., 2013), 2.6–3.7 nm (Gui et al., 2014b), 3.5 nm (Gui et al., 2014a)], which are very suitable for bioimaging applications.

For example, in 2012 Wang's and Dai's research teams collaborated to report on NIR-II Ag_2_S quantum dots with a fluorescence of 1,200 nm. The solubility of Ag_2_S QDs was changed by using a surface modification of water-soluble PEG molecules. And then Ag_2_S QDs can be dissolved in water. Ag_2_S was first used in NIR-II cell imaging and non-specific tumor detection (Hong et al., 2012b). Additionally, in 2012, they applied PEG modified Ag_2_S quantum dots with high quantum efficiency to NIR-II imaging *in vivo* (Zhang et al., 2012), using nude mice inoculated with 4T1 tumors. Not only did the Ag_2_S quantum dots were proven to be a good NIR-II fluorescence contrast agent, but interestingly, after injection into the tail of nude mice, Ag_2_S quantum dots gradually accumulated in the tumors of nude mice as the circulation time increases. They believe that the reason for above observation is the non-specific enhanced permeability and retention effect (EPR effect) of cancer tissue. Subsequently, Wang's team did an in-depth study on the long period cytotoxicity of Ag_2_S quantum dots *in vivo* (Zhang et al., 2013). The reticuloendothelial system (RES), such as liver and spleen, is the main accumulation site of Ag_2_S QDs *in vivo*, but it can be gradually metabolized or excreted over time. Additionally, the results of the blood biochemical analysis and histological examination of the rats given the Ag_2_S quantum dots for 2 months showed that Ag_2_S quantum dots had no obvious toxicity.

### Nanoparticle Alloys

In materials science, mixing various metal elements to form intermetallic compounds or alloys can greatly expand the properties of metals. Binary or ternary metal nanoparticles (or nanoalloys) can synthesize intermetallic compounds with controllable properties and structures on a nanoscale, which has attracted extensive attention from researchers (Ferrando et al., 2008). The main reason why alloy nanoparticles are fascinating is that the chemical and physical properties of alloy materials change with changes in composition, atom distribution, and particle size.

Researchers who have devoted themselves to in-depth studies of these metal nanoalloys have found that metal alloy materials not only have excellent catalytic properties, but also have excellent fluorescence properties. Further research shows that these fluorescent alloy nanoparticles inherit many advantages of the original metal nanoparticles, such as fluorescence properties, water solubility, and biocompatibility. They also can adjust the optical properties of the main metal nanoparticles or develop new functions through the introduction of another metal. For example, in 2013, Millstone's team (Andolina et al., 2013) introduced copper into fluorescent Au nanodots to form Au/Cu alloy nanodots. By adjusting the content of copper in the alloy nanodots, the fluorescence of Au/Cu alloy nanodots gradually shifted from NIR-I to NIR-II. Their group further introduced the Co element into fluorescent Au nanodots to prepare Au/Co alloy nanodots, which had both magnetic and near infrared fluorescence tunable functions (Marbella et al., 2014). Compared to the traditional near infrared quantum dots, the multi-functional alloy nanodots with near infrared fluorescence not only free of toxic elements, such as heavy metals, but also have many functions in the same material. These mutli-functional alloy nanodots have wide prospects for application in bioimaging, especially in the field of multi-mode imaging.

### Down-Conversion Rare Earth Nanoparticles

Recent research results have shown that rare earth nanomaterials have the ability of down-conversion luminescence, which is, when near-infrared light (980 nm) is used as excitation light to irradiate nanoparticles, its emission light is in the NIR-II spectrum range (Tan et al., 2009). The structure of these nano-materials is very similar to that of up-conversion nano-particles. Nanocrystals such as lanthanide nanocrystals (NaYF_4_) are used as primary materials, which doped with lanthanide elements such as Ho^3+^, Tm^3+^, and Nd^3+^ (Naczynski et al., 2013; van Saders et al., 2013). Using the basic structure of rare earth nanocrystals, multi-shell complex core-shell rare earth nanoparticles have been developed. For example, in 2013, Moghe team reported on a core-shell structure nanoparticle of NaYF_4_ which had been regenerated on the surface of existing NaYF_4_ Yb:Ln nanocrystals (Naczynski et al., 2013). By changing the doped lanthanides (Er, Ho, Tm, Pr), NIR-II fluorescence with different wavelengths can be obtained under 800 nm excitation, and the longest wavelength can reach up to 1,500 nm. Compared with up-conversion rare earth nanoparticles, down-conversion nanoparticles have received less attention, but they are still widely applied *in vivo* bioimaging research (Naczynski et al., 2013, 2014; Jiang et al., 2016). Li et al. reported on a dual-function particle system using up-conversion and down-conversion (Li et al., 2013), where the particle can emit visible (800 nm) and near-infrared light (980 nm) according to the excitation light of different wavelengths and can be used in biological imaging.

In recent years, it has been found that Nd ion not only has NIR-II fluorescence emission with wavelength of 1,050 or 1,300 nm, but also has the sensitization effect on Ytterbium ion. It is excited at a biocompatibility wavelength below 800 nm, which has the lowest absorption of water. Nd^3+^ has been recognized gradually as a photosensitizer (Hemmer et al., 2016), and Nd^3+^ doped near infrared fluorescent nanoparticles have attracted great interest from researchers who study lanthanide-doped biological probes. One kind of Nd^3+^ doped near infrared fluorescent materials is an NaYF_4_ type down-conversion lanthanide nanocrystal (Chen et al., 2012), and the other is an Nd^3+^ doped fluoride nanoparticle (Pokhrela et al., 2014). For example, in 2015, Garca's group synthesized Nd^3+^ doped SrF_2_ nanoparticles and used them reduce background fluorescence (Villa et al., 2015), since minimizing any background fluorescence is essential for high contrast bioimaging. The excitation wavelength of the nanoparticles is 808 nm, and the emission wavelength is 1,100 nm, which belongs to NIR-II fluorescence. The nanoparticles are mixed into the feed and fed to mice. After the nanoparticles enter the mice through the digestive system, *in vivo* fluorescence imaging is performed. It was found that an NIR-II fluorescence of 1,300 nm generated by the ^4^F_3/2_-^4^I_13/2_ orbital transition of Nd^3+^ effectively eliminated the background fluorescence. It can realize the NIR-II imaging of deeper structure, inorganic spontaneous fluorescence and high distinguishability *in vivo*. In addition, in 2016 Prasad's team prepared a hybrid organic-inorganic system to form an epitaxy of NaYF_4_: Yb^3+^/X^3+^@NaYbF_4_@NaYF_4_:Nd^3+^ (X = null, Er, Ho, Tm or Pr) core/shell/shell (CSS) nanocrystals, and coated ICG on the external layer of CSS nanocrystals. This hybrid system can capture NIR light in a wide excitation spectrum range (700–860 nm) by ICG and produce effective polychromatic narrow-band NIR-II emission light from 1,000 to 1,600 nm according to the different doping elements in the nucleus. Further experiments show that the NIR-II emission fluorescence can be used to image clearly at a tissue depth of 9 mm and detect optical signal at a tissue depth of 23 mm (Shao et al., 2016).

### Nanoparticles Based on Organic Fluorescent Dyes

Until now, organic fluorescent dyes are still the most widely used luminescent markers in fluorescence imaging. Organic fluorescent dyes have attracted much attention due to their high fluorescence quantum efficiency, easy functionalization, and adjustable luminescence spectra (Thekkek and Richards-Kortum, 2008; Willmann et al., 2008; Kobayashi et al., 2010; Sinkeldam et al., 2010). The commonly used organic fluorescent dyes presently include naphthalimides, coumarins, fluoresceins, rhodamine, anthocyanins, BODIPY (Lu et al., 2014), porphyrin phthalocyanine, and other macrocyclic molecules. The absorption and emission wavelengths of these commonly used organic fluorescent dyes cover the ultraviolet, visible, and near infrared regions (Mishra et al., 2000; Lavis and Raines, 2008; Ma and Su, 2010).

**Figure 2:**
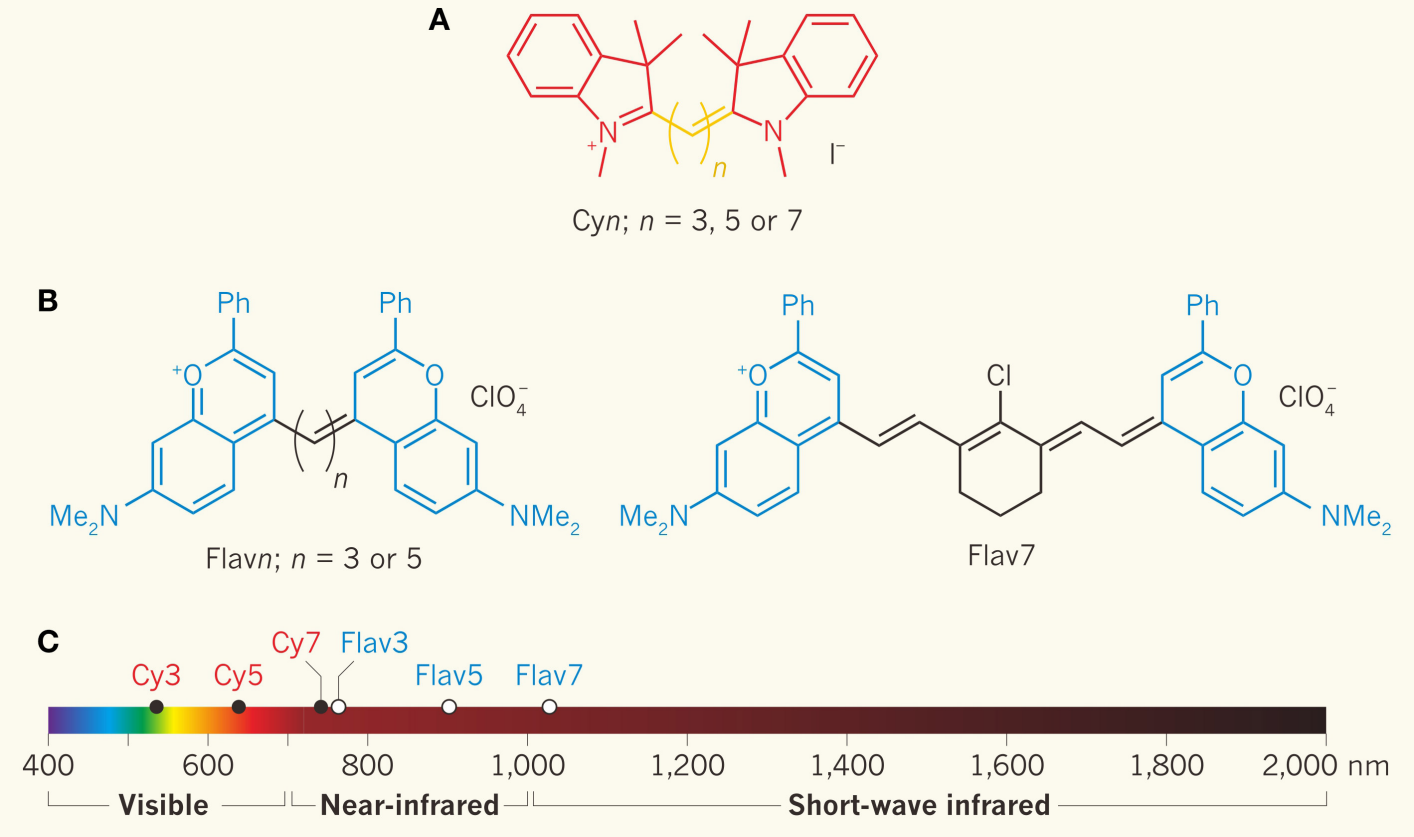
Increasing the length of polymethine chain was proved to result in the red shift of cyanine dye's emission. Cosco et al. developed new methods of heterocyclic conjugation and added new electron donor groups (Cosco et al., 2017; Zhu et al., 2018). **(A)** Molecular structure of cyanine dye series of compounds for optical fluorescence imaging. **(B)** The blue structure is the compound of dimethyl-flavylium heterocycles, which can be used to replace the indolenines to prepare flavylium polymethine fluorophores. **(C)** The emission and absorption of Flav7, the modified organic small molecule fluorescent dye, was in the NIR-II window. Adapted from Cosco et al. (2017) and Schnermann (2017) with permission.

Over the years, how to convert NIR-I fluorescent probes directly into NIR-II fluorescent probes by molecular engineering methods and design principles has been a highly discussed topic among investigators. According to the available literature (Yang et al., 2017; Zhu et al., 2018, 2019; Wu et al., 2019), the specific design principles are as follows: (1) The typical structure of NIR-II small molecule fluorescent dyes is a molecular structure composed of donor-acceptor-donor (D-A-D) (e.g., CH1055-PEG), which is modified to form a class of dyes with similar properties and emission wavelengths. The specific emission principle uses aromatic π-bridge connectors as molecular wiring. These electron-supplying groups can produce enhanced electron shifts and low energy gaps to the central electron acceptor, resulting with the dye molecule having the capability of NIR-II emission. (2) By systematically adjusting the electronic donor part, the π-bridge connector and the functional groups at the end of the fluorescent dye molecule promote quantum yield. Specific strategies to improve the brightness of small molecular dyes include enhancing intramolecular charge transfer and molecular stiffness, creating complexes with serum proteins, such as human serum albumin (HSA), and introducing a protecting group (S) to produce a S-D-A-D-S structure at the end of the dye, which protects the dye's pillars from intermolecular interactions and fluorescence quenching polymerization. (3) By increasing the conjugate length to separate electron donor/acceptor and heteroatom substitution, the fluorescence emission of existing polymethine dyes (i.e., cyanine dye) can be re-shifted to produce a new fluorescent dye that can emit NIR-II fluorescence with high quantum yield and absorption coefficient (ε) (Figure 2). At present, polymethine molecular dyes with emission wavelength more than 1,000 nm are available on the market, including IR-1040, IR-1048, IR-1051, and IR-1061. Unlike the previous method of for red-shifting anthocyanin dyes by simply increasing the length of polymethine chain, by Cosco et al. (2017) propose of a new method of extending heterocyclic conjugation and adding new electron donor groups. This new method has been proven as a feasible method for red-shifting anthocyanin dyes. In addition, most of the NIR-II small molecule fluorescent dyes mentioned above are organic products with low water solubility, which need to be encapsulated in a hydrophilic matrix to enhance biocompatibility *in vivo* imaging. Recently, Li et al. (2018) developed a novel small molecule fluorescent dye FD-1080 through a structural redesign of a typical anthocyanin dye. Its excitation and emission wavelength are both in the range of NIR-II. Besides there are reports about indocyanine green (ICG). ICG is a NIR fluorescent dye with strong absorption, low toxicity, no involvement in biological transformation *in vivo* and rapid excretion. It is the only NIR optical imaging contrast agent approved by the US Food and Drug Administration (FDA) for clinical use. Because the absorption and fluorescence spectra of ICG are in the NIR-I window (650–950 nm), ICG has been widely used in cardiovascular system, liver function evaluation, visualization of retina and choroid, ophthalmic angiography, cerebral angiography, and other clinical fields. Recently Starosolski et al. (2017) found that ICG dye also has NIR-II fluorescence characteristics in the NIR-II window. This discovery has opened up new uses of ICG and will greatly expand the application scope of ICG. In the experiment, the absorbance and NIR-II fluorescence emission of ICG were detected in different concentrations of ICG media, including PBS, plasma and ethanol. The customized spectral NIR module is used for *in vitro* and *in vivo* tests, and the images of NIR-I and NIR-II windows are obtained at the same time. The results show that ICG has a significant fluorescence emission in the NIR-II window at the wavelength of about 1,100 nm, and this emission (similar to the absorption curve) is essentially affected by the molecular environment of ICG. The results of *in vivo* imaging further illustrated that ICG can be used as NIR-II fluorescent dye and the contrast-to-noise ratios (CNR) value was twice that in NIR-I window. Clinical transformation of NIR-II imaging technology can be accelerated when ICG, an FDA approved imaging agent, is used. Later, the research team published another paper on NIR-II fluorescence imaging using indocyanine green nanoparticles (Bhavane et al., 2018). In this study, they collected the fluorescence spectra of ICG liposomes in PBS and plasma. *In vivo* imaging research was carried out to observe the vascular structure of the hind limbs and the intracranial region in real time. Free ICG, NIR-I imaging and cross-sectional imaging (MRI and CT) were used as controls. The results showed that the liposome ICG had strong NIR-II fluorescence, similar to the free ICG in plasma. *In vitro* studies have shown that liposome ICG has better performance than free ICG in the NIR-II imaging of deep (≥4 mm) vascular analog structure. *In vivo*, NIR-II fluorescence imaging with liposome ICG can significantly improve the contrast (*P* < 0.05) compared with long-term free ICG, and make the hind limbs and intracranial vessels visualized within 4 h after injection. Compared with NIR-I imaging, liposome ICG enhanced NIR-II imaging has better vascular clarity. Subsequently, several research papers on tumor imaging using Second-Window-ICG (SWIG) technique were published, and this field has become the latest research hotspot of NIR-II imaging (Zeh et al., 2017; Cho et al., 2019a,b; Suo et al., 2019; Wang et al., 2019b).

## Applications of NIR-II Window Bioimaging in Biomedicine

### Tumor Imaging and Image-Guided Surgery

Solid tumor patients often fail in treatment due to local recurrence. Cytopenic surgery is targeted to improve staging and reduce tumors using fluorescence imaging to guide the specific resection of tumors and significantly improve the prognosis. Recently, this application field has become a high-interest topic in research, resulting in the publishing of many high-level papers.

Papers published by Wang et al. (2018) in Nature Communications, for example, report on a novel down conversion nanoparticle emitted in the NIR-II window for living assembly. In metastatic ovarian cancer, fluorescent nanoprobes modified with DNA and targeted polypeptides are used to ensure the precision of cytoreduction surgery. The image distinguishability of NIR-II nanoparticles is better than that of clinically recognized ICG, mainly due to its better light resistance and deeper tissue penetration (8 mm). After the nanoprobe is assembled *in vivo*, the stable preservation time of the nanoprobe on tumors is up to 6 h, so the nanoprobe can be used in precise tumor resection surgeries. The probe successfully obtained a superior ratio of tumors to normal tissues, which can help to delineate the resection profile in the surgery for metastatic abdominal ovarian cancer. The results also showed that metastases <1 mm could be completely removed under the guidance of NIR-II bioimaging. This novel method provides a new idea for the design of nanomaterials for medical applications in the future.

**Figure 3:**
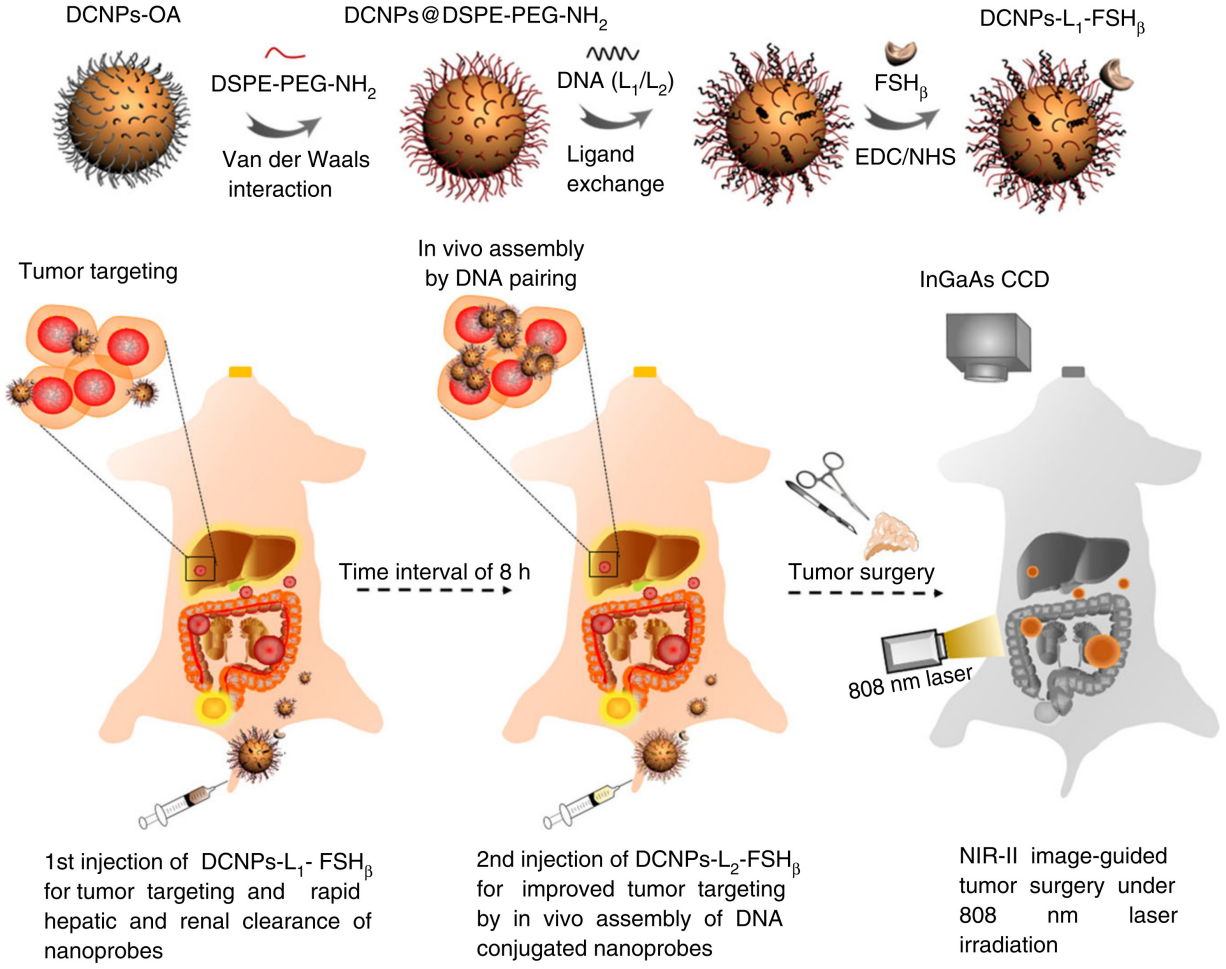
Construction of NIR-II nanoprobe schematic diagram in surgery for metastatic ovarian cancer guided by NIR-II bioimaging (Wang et al., 2018). The diagram above shows the preparation of DCNPs (DCNPs-L1-FSH_β_) modified by DNA and FSH_β_. The schematic diagram below shows the further assembly of the NIR-II nanoprobe *in vivo* by a two-step sequential injection of DCNPs-L1-FSH_β_ (the first injection) and DCNPs-L2-FSH_β_ (the second injection), which is beneficial to improve tumor targeting and rapid liver and kidney clearance. Under the guidance of NIR-II imaging, metastatic ovarian tumors can be clearly observed and accurately removed. Adapted from Wang et al. (2018) written by Fan Zhang etc. with permission.

The key to the implementation of above-mentioned study is the *in vivo* assembly of novel NIR-II fluorescent nanoparticles, which is also the ingenuity of this research design. The specific assembly principle is shown in Figure 3. At the top of Figure 3 is a schematic of the step-by-step construction of DNA and FSH_β_ modified down-conversion nanoparticles (DCNPs-L_1_-FSH_β_). The core of the nanoprobe are lanthanide doped core-shell nanoparticles, consisting of a 5.0 nm luminescent gadolinium tetrafluoride sodium doped 5% neodymium (NaGdF_4_:5% Nd) encapsulated by a 2.5 nm thick homogeneous gadolinium sodium inert shell. This type of a structure can avoid fluorescence quenching by water and bind complementary DNA (L_1_ or L_2_) on its surface. FSH_β_ is a follicle stimulating hormone peptide that specifically binds to ovarian cancer epithelium to enhance the targeting efficiency of the fluorescent nanoprobe. Because of the great potential of DNA complementary strand hybridization, the nanoprobe can be stable hybridized on the surface of tumors *in vivo* for bioimaging. Next, a process diagram of the fluorescent nanoprobe assembly *in vivo* is presented in the lower part of Figure 3. It greatly enhances the tumor targeting of the probe, but also enables the probe to be excluded from the body by the liver and kidney more quickly after the operation. The specific process is to implement two-step injection of DCNPs-L_1_-FSH_β_ and DCNPs-L_2_-FSH_β_, in succession. In order to verify the performance of two-step *in vivo* simultaneous assembly of fluorescent nanoprobes, the fluorescent dye Cy5 was grafted into the probe assembly structure of the first needle, i.e., DCNPs-L_1_-(Cy5)-FSH_β_, and Cy7 were combined to the probe assembly structure of the second needle, i.e., DCNPs-L_2_-(Cy7)-FSH_β_. When the complementary DNA fragments L1 and L2 in the two components of the probe are hybridized close to each other, the two dyes will cause fluorescence resonance transfer (FRET), which is more than twice as strong as two dyes without FRET. The results show that the two components of the probe can be specifically assembled on the surface of tumors *in vivo*. Ultimately, surgery guided by NIR-II imaging can clearly observe metastatic ovarian cancer and precisely resection it.

Other recent representative work includes the development of Ag_2_Te quantum dots by Zhang et al. (2019), which can emit fluorescence at 1,300 nm wavelength after assembly. The assembly process is divided into two steps; first, polymerization is conducted with polylactic-co-glycolic acid (PLGA), and second, further packaging is completed with the cancer cell membrane. The fabricated biomimetic nano-biological probes have a bright and highly stable fluorescence in the NIR-II window. Through the second step of surface encapsulation of active homologous tumor membranes, the probe enhances the ability to target tumors. Tumor targeting occurs through the passive enhancement of permeability and retention of homologous cancer cell membranes, which can increase the accumulation of probes in the tumor sites. Zhang et al. successfully prepared a novel biomimetic NIR-II fluorescence nanoprobe which showed characteristics of ultra-bright, stable fluorescence, homologous targeting and high biocompatibility, which can significantly enhance the imaging of living tumors. In 2018, Yang et al. (2018) reported on a NIR-II lanthanide complex (Nd-DOTA) probe that can be rapidly excreted. Within 3 h after injection, more than 50% of the probe can be excreted through the kidney. The molecular weight of the probe is only 0.54 KDa, and in terms of light resistance and tissue penetration, the NIR-II imaging quality of the new probe is much better than that of the clinically approved ICG. Yang et al. also demonstrated the ability to obtain an excellent tumor-to-normal tissue ratio, which can help to accurately mark the profile of micro-tumors in surgery for abdominal metastatic ovarian cancer. Metastatic tumors <1 mm can be completely removed under the guidance of NIR-II bioimaging. In addition, the probe Nd-DOTA has the same structure as Gd-DOTA, and Gd-DOTA is a clinically recognized MRI contrast agent. Therefore, it may be a straightforward path for the clinical transformation of the new NIR-II probe. In 2018, Shou et al. (2018) prepared a semiconductor polymer nanoparticle of diketopyrrolopyrrole (PDFT1032) and developed it as a NIR-II (near infrared window 1,000–1,700 nm) fluorescent probe for *in vivo* tumor imaging and image-guided tumor re-sectioning. The NIR-II probe has many advantages, including stable fluorescence emission, high absorption efficiency at 809 nm, large Stokes shifting, biocompatibility, and lower toxicity *in vivo*. Moreover, research has shown this NIR-II probe has a wide range of applications in the biomedical field. For example, tumor imaging in terms of subcutaneous osteosarcoma patterns, evaluating vascular embolization treatment on tumors, *in-situ* cytoreduction surgery guided by NIR-II images, and sentinel lymph node biopsy with superior temporal-spatial resolution (SLNB). In general, PDFT1032 not only has good biocompatibility and hydrophilicity, but also excellent chemical and optical properties. The NIR-II fluorescent probe has a wide application prospect in the imaging of malignant tumors and cytoreduction surgery.

### Medical Testing

Fluorescence bioimaging can detect deep tissues in the NIR-II window, and it has the smallest self-fluorescence and tissue scattering. Nevertheless, *in vivo* NIR-II fluorophores detection is merely concentrated on direct disease lesions or living organ imaging, and producing real-time dynamic NIR-II fluorescence biosensor has been challenging. In recent years, new methods and breakthroughs have been emerging in this field, such as the conception and progress of quantitative detection with fluorescent probe of ratio meter.

**Figure 4:**
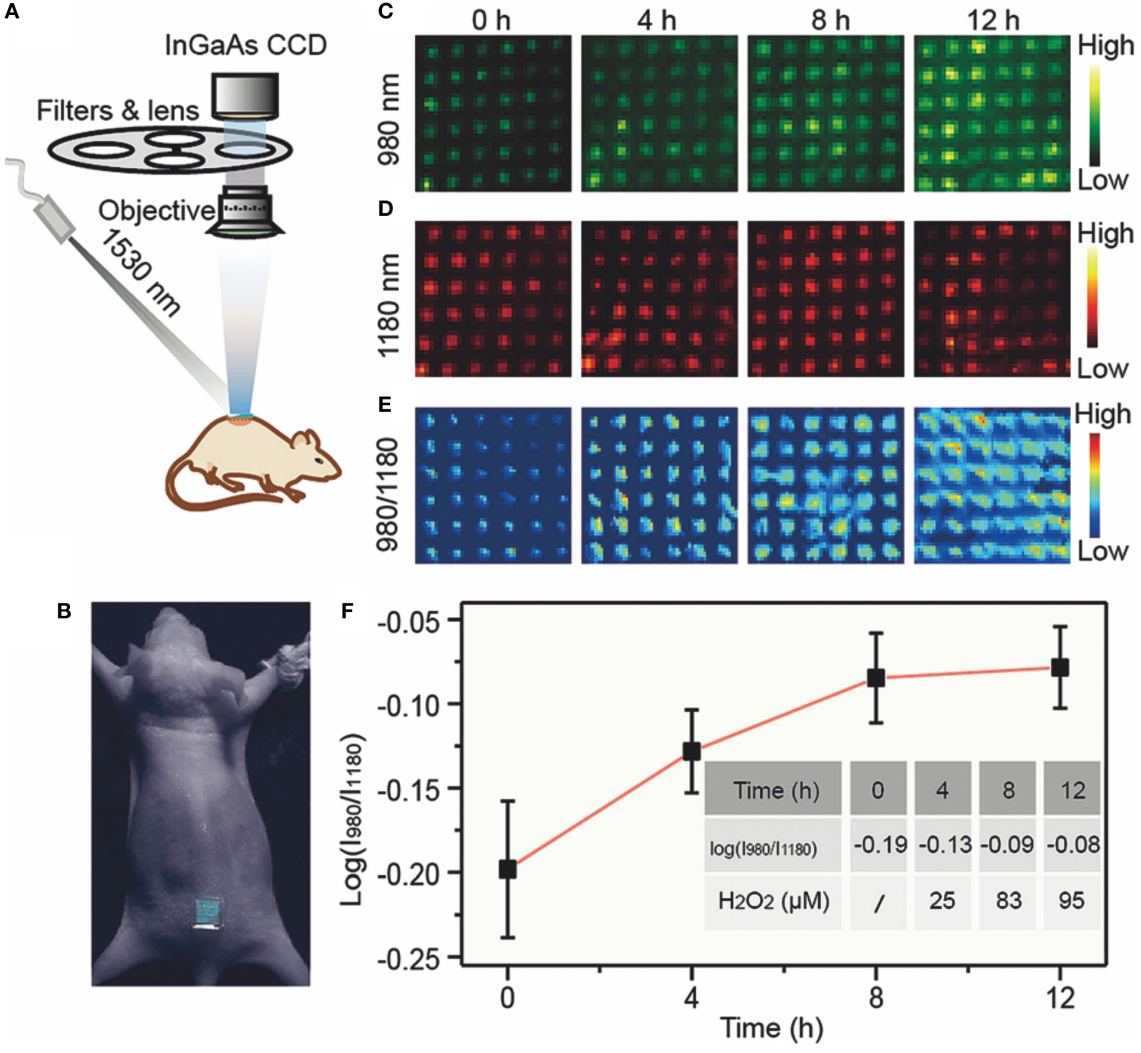
Operational diagram of ratiometer fluorescence sensor (Liu et al., 2018). **(A)** *In vivo* bioimaging experimental apparatus. **(B)** Pictures of mice treated with microneedle patches. After lipopolysaccharide was used to induce inflammation in mice, the upconversion luminescent images of microneedle patch at 980 nm **(C)**, 1,180 nm **(D)**, and ratio (I_980_/I_1180_) **(E)** channels were detected at different times. **(F)** Ratiometric fluorescence (I_980_/I_1180_) of microneedle patches at different time and corresponding H_2_O_2_ concentration. Adapted from Liu et al. (2018) written by Fan Zhang etc. with permission.

In Fan Zhang's paper (Liu et al., 2018) published in Angewandte Chemie International Edition in 2018, he reports a novel Erbium-sensitized up-conversion of nanoparticles, which have the characteristics of both 1,530 nm excitation and 1,180 nm emission in the NIR-II window, which can be used *in vivo* biosensors. This research studies a micro-manipulation needle debris sensor for *in vivo* inspection of inflammation in real time, where the detection principle of the sensor is based on the ratio meter fluorescence combined with efficient NIR-II fluorescent emission and the organic probe sensitive to hydrogen peroxide under the action of Fenton catalyst Fe^2+^. Finally, the dynamic detection of inflammation *in vivo* is successfully produced. This NIR-II radiometric probe has the following advantages: large anti-Stokes displacement, low background fluorescence, low absorption, and scattering in biological tissues. Therefore, the probe can detect and evaluate inflammation *in vivo* in real time with high imaging quality. The specific process of *in vivo* inspection of inflammation in real time by the fluorescence sensor of the ratio meter is shown in Figure 4, including the device diagram of fluorescence probe used for confirmatory testing *in vivo*, the fluorescence value of the ratio meter (I_980_/I_1180_) channel at different times, the quantitative curve of ratiometer fluorescence (I_980_/I_1180_), and the corresponding concentration of hydrogen dioxide at different times.

Figure 4A is the device diagram of the ratiometer fluorescent probe used for confirmatory testing *in vivo*, and Figure 4B is a photograph of a mouse treated with fragments of a micromanipulator needle. Figures 4C–E show the 980, 1,180, and ratio gauge (I_980_/I_1180_) channels, respectively. The micromanipulator needle fragments obtained fluorescence values at different times after LPS-induced inflammation. Figure 4F is a quantitative curve of the ratio meter fluorescence (I_980_/I_1180_) of the micromanipulation needle fragments and the corresponding concentration of hydrogen peroxide at different times. It can be used to quantify the degree of actual inflammation in clinical tests.

Other recent studies in this field include Zhao et al. (2019) which reported on an original method for the accurate imaging of inflammation *in vivo* using *in situ* cross bonding of glutathione-combined ultrafine lanthanum nanoparticles with NIR-II fluorescent emission. Although nanoprobes have been proven to be promising bioimaging platforms by reason of their EPR effects, to the inability to enrich nanoprobe at target position has been a key bottleneck to improve detection ability and effect. While cross bonding of nanoparticles *in vivo* can increase the enrichment of EPR region (e.g., inflammatory areas), nanoparticles are absorbed by RES, resulting in unidirectional cross bonding in non-target organs. Based on these difficulties, the principle of this strategy was developed to enhance the *in vivo* imaging by using sub-10 nanometer glutathione (GSH) combined lanthanide nanoparticles, which react with reactive oxygen species (ROS) in the inflammation region to locate and image reactive oxygen species rapidly in the NIR-II window. At the same time, these nanoprobes can be excreted quickly because of their ultrafine magnitude. Based on the *in-situ* crosslinking and rapid excretion ability of the probe, this method can achieve accurate biological imaging and is suitable for other ultrafine contrast agents.

In 2019, Wang et al. (2019a) developed a reverse quenched NIR-II molecular fluorophore and applied it to high contrast imaging and pH sensing *in vivo*. Currently, the molecular fluorophore with contrast and sensitivity in NIR-II window (1,000–1,700 nm) have both been developed *in vivo* fluorescence imaging. However, the solvation of long-wavelength absorbed fluorophore in aqueous solution, results in quenching, which is challenging to avoid. Therefore, a series of reverse-quenched pentamethine cyanine fluorophore have been developed, which significantly overcome the serious solvation coloration, thus providing a stable absorption/emission of fluorescence over 1,000 nm in aqueous solution. The fluorescence intensity increases up to ~44 times and has excellent photostability. The conditions for lymphatic imaging can be met thanks to these advantages, including the need for tissue penetration up to 8 mm, high definition, and optical stability. Its imaging effect is better than the clinically approved indocyanine green. In addition, the fluorophore display pH-responsive fluorescence allows for non-invasive ratio meter fluorescence imaging and gastric pH quantification *in vivo*. The results show that this method is consistently accurate when tissue depth is 4 mm compared to the standard pH electrode method. This work opens up the potential of reverse quenching pentamethylene cyanine in NIR-II biological applications.

In 2019, Li et al. (2019c) developed a NIR-II fluorescence molecular probe activated by peroxynitrate for drug-induced hepatotoxicity detection. Drug-generated liver injury is a key problem to the safety of drug research and use. The emergence of peroxynitrite (ONOO^−^) is considered to be an initial signal of drug-generated liver injury. Therefore, the structure of the fluorescent probe is designed as the combination of benzothiophenacyl cyanine skeleton and phenyl borate group. The NIR-II fluorescence of this probe can be activated by ONOO^−^ and can detect ONOO^−^ sensitively. When the probe IRBTP-B and the target ONOO^−^ exist at the same time, the structure of probe changes to produce the fluorescent group IRBTP-O to turn on the NIR-II fluorescence. The linear relationship between the NIR-II fluorescence intensity and the concentration of ONOO^−^ is acceptable. Tissue model studies confirm that reliable activation signals can be obtained at penetration depths up to 5 mm. With this probe, the up-regulation of ONOO^−^ in the model of incubation period drug-generated hepatotoxicity and the repair effect of N-acetylcysteine (NAC) *in vivo* could be seen. In conclusion, this method will be used as a general method to develop activated NIR-II probes triggered by specific analytes based on hydroxyl functionalization reaction sites.

### Vascular Biological Imaging

The number of people with cardiovascular and cerebrovascular diseases has increased, as has the ability to detect these diseases using examination technology, which, in turn, has led to an increase in incidence of cardiovascular and cerebrovascular diseases. The commonly used techniques in clinical angiography include B-mode ultrasonography, CT scans, and nuclear magnetic resonance (NMR). However, these techniques are non-tumor-specific, have low-sensitivity and high-cost, and can't be used for real-time detection or clinical practice in surgical operations. In recent years, some research groups have designed NIR-II fluorescence probes and injected them into mice intravenously, after which the NIR-II fluorescence is used to perform vascular imaging *in vivo*. The results showed that the NIR-II fluorescence could penetrate the skull of mice to a deeper level, not only imaging the distribution of blood vessels in the heads of mice, but also clearly showing the fine structure of capillaries.

**Figure 5:**
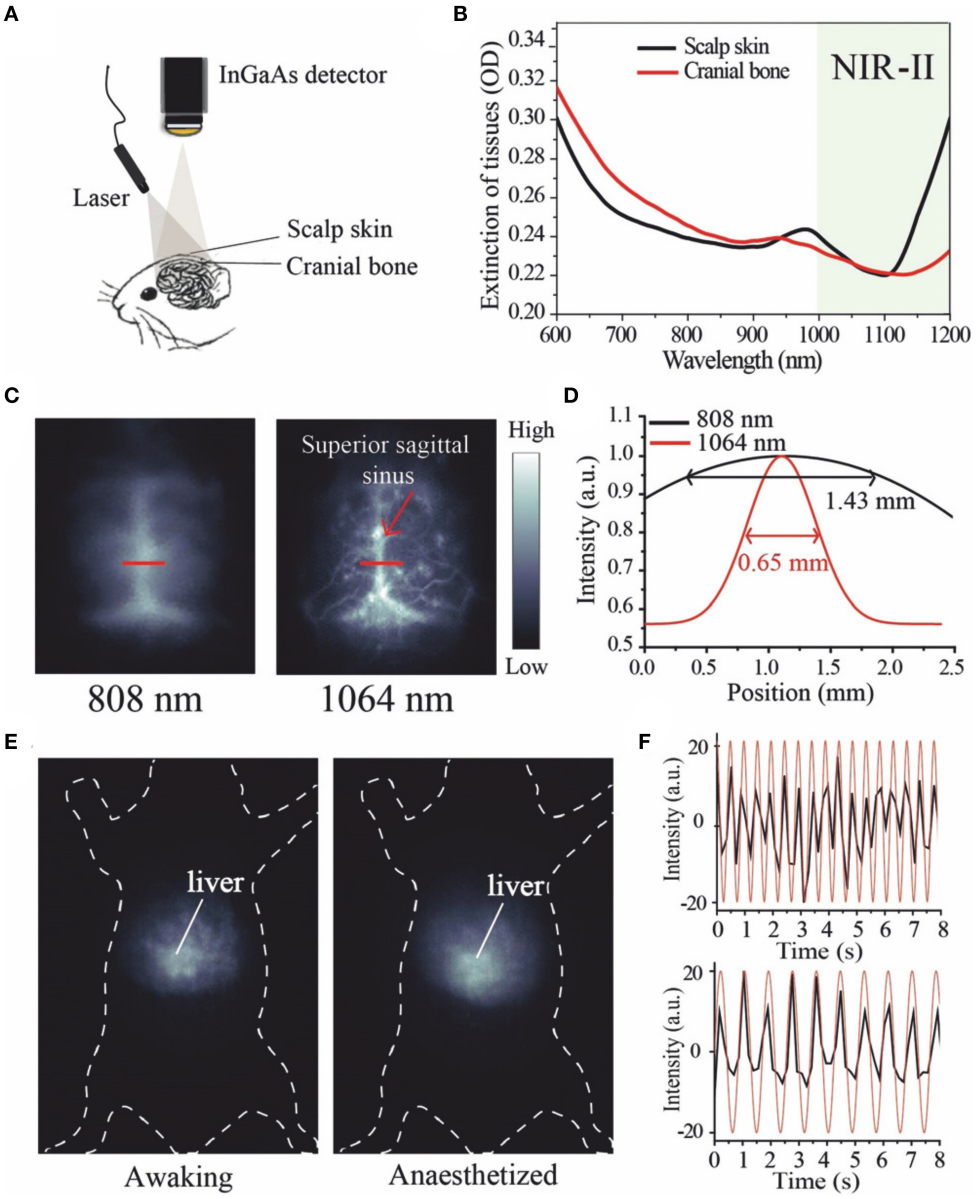
Fluorescence imaging of rat brain vascular system and breathing rate of mice using NIR-II fluorescence probe FD-1080 (Li et al., 2018). **(A)** Schematic diagram of NIR-II optical imaging through brain tissue scalp and cranial bone. **(B)** Extinction spectra of scalp skin and skull. The black curve represents the scalp skin and the red curve represents the skull. **(C)** Fluorescence images of FD-1080-FBS complex were compared under different excitation conditions as indicated. **(D)** The fluorescence intensity profile fitted by gaussian was distributed on a red line of interest, with excitation wavelengths of 808 and 1,064 nm, respectively. **(E)** The distinct emission of the FD-1080-FBS complex made the awake and anesthetized mice imaged, and under the excitation of 1,064 nm detected the signal fluctuations generated by the liver movement. **(F)** Respiratory rates in awake (upper) and anesthetized (lower) mice. Adapted from Li et al. (2018) written by Fan Zhang etc. with permission.

In 2018, Li et al. (2018) published papers within this field of study in the Angewandte Chemie, regarding the successful synthetization of a small molecule fluorescent probe FD-1080, which has excitation and emission both in the NIR-II region and it has excellent bioimaging capability of the vascular system *in vivo*. The structure of heptamethine in fluorescent probe FD-1080 has been ingeniously designed as a key group for the conversion of absorption and emission into the NIR-II region. An acidic sulfuric acid group and cyclohexene group were applied to intensify the water dissolvability and chemical durability of the probe. The quantum yield of FD-1080 was 0.31% and improved to 5.94% when combined with bovine fetal serum (FBS). It is noteworthy that compared with the excitation wavelength from 650 to 980 nm in NIR-I region in the previous work, the excitation wavelength at 1,064 nm in NIR-II region has proved to have stronger penetration in biological media and excellent imaging quality. FD-1080 can not only achieve non-invasive, high-resolution, in-depth tissue bioimaging of the vascular and cerebrovascular systems, but it also quantifies respiratory frequencies of awake and anesthetized mice according to dynamic images of respiratory craniocaudal motion within the liver. The key steps in this study included the schematic diagram of the vascular imaging device, the fluorescence imaging of the rat cerebral vascular system with the NIR-II fluorescence probe FD-1080, and the breathing rate detection graph of the rat, as shown in Figure 5.

Figure 5A is a device diagram of NIR-II fluorescence penetrating the scalp and skull brain tissue for an angiography. Figure 5B is the disappearance spectra of the scalp (black) and skull (red). It has been found that the strongest penetration is at 1,064 nm. Figure 5C contrasts the fluorescence images of FD-1080-FBS complex in a rat brain vasculature system under different excitation lengths (1,300 long pass filter), where it was found that the clearest angiogram was at the wavelength of 1,064 nm in NIR-II region. In Figure 5D, a red line of interest is marked at the Gaussian fitting fluorescence intensity profile at the 808 and 1,064 nm excitation wavelengths, respectively. It is obvious that at 1,064 nm the fluorescence intensity is higher and the peak is narrower. When stimulated at 1,064 nm (1,300 nm long pass filter), the clear emission of the FD-1080-FBS complex enables the imaging of an alert or anesthetized mouse to detect the signal fluctuations produced by liver movement (Figure 5E). Figure 5F is a spectrogram of the breathing rate of alert (above) and anesthetized (below) anesthetized mice. The graph shows that there are obvious differences in the spectrogram of breathing rate between the two groups of mice, which can be used to quantify the respiratory rate of mice under different conditions.

Other studies have also been recently conducted in this field, including Li et al. (2019a) in 2019, who developed a rare earth nanoprobe triggered by 808 nm laser, which emitted NIR-II fluorescence for small tumor detection and angiography. In this study, polyacrylic acid (PAA) modified sodium tetrafluorolutetium: gadolinium/neodymium nanorods (PAA-NRs) were prepared into single crystal hexagonal phase and unified magnitude, and then further developed as a highly sensitive NIR-II imaging probe for optical imaging navigated detection of tiny tumors, angiogenesis-related diseases, and angiogenesis diagnosis. The NIR-II emission wavelength with sodium tetrafluorolutetium: gadolinium as the main body can be easily adjusted by changing neodymium doping, which makes it hopeful that the emission center will have high optical stability at 1,056 and 1,328 nm. The probe has a high spatial resolution (~105 μm) for small blood vessels *in vivo* and can detect them clearly. The *in vivo* tracking experiments with time changes confirmed that PAA-NRs probe was mostly collected in the RES, and discharged from the body by the liver. Histological examination revealed that the hydrophilic nanorods had very low toxicity and great biological compatibility in living animals.

In the same year, Li et al. (2019b) reported a polydopamine-coated multifunctional lanthanide diagnostic agent, which can be used for angiography of vascular malformations and tumors, as well as photothermal therapy guided by imaging at wavelengths above 1,500 nm. NIR-II optical imaging with emission wavelengths >1,500 nm can be used as the next generation fluorescence imaging technology to guide the display technology of tumor vessels and vascular malformations, which can then be used for early tumor diagnosis and recognition of tumor-related vascular characteristics. This study is based on the core-shell structure of NaLuF_4_ nanorods@polydopamine (NRs@PDA), which combines advanced NIR-II fluorescence imaging ultra 1,500 nm wavelength and photothermal (PTT) functions. This is a high-performance integrated nano-platform for diagnosis and treatment developed for visualization of tumor-related vascular malformations and imaging-guided photothermal therapy.

In 2018, Wan et al. (2018) developed a bright organic NIR-II fluorescent nanocluster (p-FE) for three-dimensional imaging of biological tissues, which can provide bright NIR-II fluorescent emission light with a wavelength >1,100 nm for non-invasive *in vivo* blood flow tracking in the rat cerebrovascular system. Moreover, p-FE can produce a layer-by-layer image of the vascular system based on single-photon and three-dimensional confocal imaging in a fixed rats brain tissue. Its depth is up to 1.3 mm, and it has a spatial resolution of up to 10 microns. The study also completed *in vivo* dual-color fluorescence imaging in the NIR-II window, using p-FE emitted between 1,100 and 1,300 nm as a vascular imaging reagent, as well as single-walled carbon nanotubes (CNTs), which emitted more than 1,500 nm as a contrast agent to highlight tumors in mice. Ultimately, the results demonstrated excellent dual imaging of vascular system and tumors.

### Diagnosis and Treatment of Cancer

Each imaging technology has its own unique advantages and inherent limitations. For example, fluorescence imaging has excellent sensitivity, but low tissue penetration and spatial resolution in turbid media. MRIs, CT scans, and ultrasound imaging have better spatial resolution, but their sensitivity is limited. The sensitivity of PET-CT scan is relatively high, but it can't provide the structural information of the imaging material. Therefore, multi-mode imaging, which integrates two or more imaging technologies, can supplement the imaging information and diagnose diseases with reliable accuracy. In order to meet the needs of multi-mode imaging, researchers usually combine fluorescent nanomaterials with functionalized small molecules or particles by doping, bonding and coating to make them multi-functional nanoparticles, which can be used in multi-mode biological imaging.

**Figure 6:**
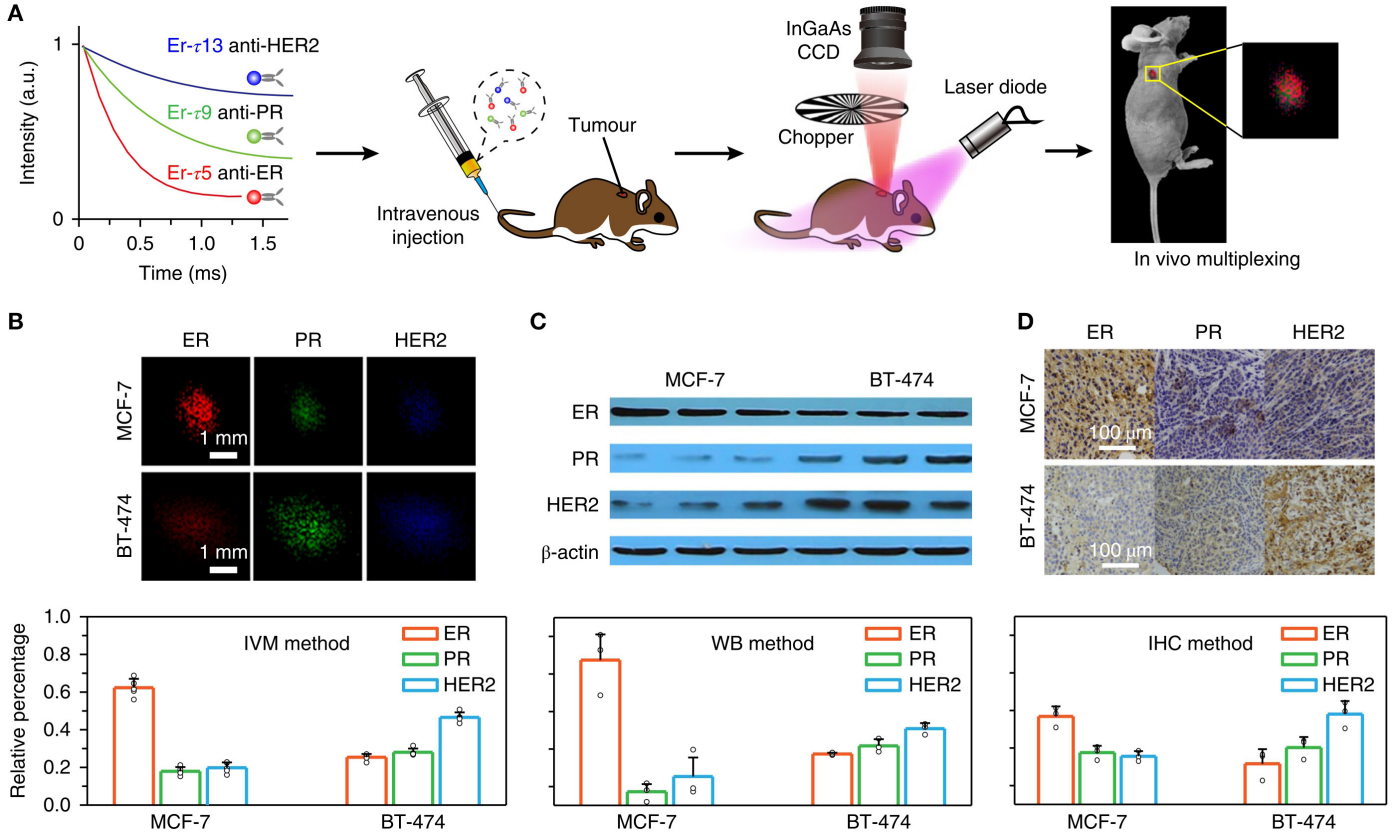
IVM technique of time-resolved imaging for tumor biomarkers (Fan et al., 2018). **(A)** A diagram showing the procedure of an animal experiment. Three groups of Er nanoparticles with different lifespans were combined with three antibodies (anti-ER, anti-PR, and anti-HER2) and were transplanted into mice via the caudal vessel. Lifespan distinguished imaging was then accomplished with IVM to quantitatively analyze biomarker expression on the tumor. **(B)** The lifespan distinguished images of McF-7 and bt-474 tumors were decomposed into three lifespan paths, which were red, green and blue monochromatic images. The pattern of biomarker expression was determined by integrating the intensities of each component and standardizing the overall intensity of the whole tumor area. Using the results of *in vitro* western blot **(C)** and *in vitro* immunohistochemistry assay **(D)**, the biomarker expression modes of IVM in two tumor hypotypes were calculated. Adapted from Fan et al. (2018) written by Fan Zhang etc. with permission.

Deep tissue imaging within the NIR-II window has great prospects in physiological research and biomedical applications. However, the inhomogeneous signal attenuation in biological material limits the application of multi-wavelength NIR-II probes in the imaging of various biomarkers and cancer diagnoses. For example, Fan et al. reported in 2018 (Fan et al., 2018), that NIR-II nanoparticles doped with lanthanides have the ability to design the luminescence time for quantitative imaging *in vivo* after using multiplexing in the time domain. To achieve this, a systematic research method based on controllable energy transfer was designed to create an adjustable lifetime range of nanoprobes, which had a luminescence time spanning three orders of magnitude and had only one emission band. When the depth of the NIR-II nanoparticle probe in biological tissue reached 8 mm, the selected nanoparticle duration was continuously resolved, and the SNR was taken from the time node when light intensity was <1.5. Powerful time-length editors have been shown to be independent of tissue penetration depth, and *in vivo* multiplexing technology (IVM) has been used to diagnose tumor hypotype in living animals. The data in this paper are in good agreement with those of a standard *ex vivo* immunohistochemistry assay, which proves that the luminescent time-length imaging can serve as a microinvasive method for tumor definite diagnosis. The research process and key points of IVM for tumor biomarkers using specific luminescence time-length imaging are shown in Figure 6.

Figure 6A illustrates the schematic diagram of conducted animal experiments. Three batches of Erbium nanoparticles were bound to three different kinds of antibodies (anti-ER, anti-PR, anti-HER2) and were transplanted into mice via the caudal vessel and resulted showed that each had a different luminescence duration. Then, time-resolved images using *in vivo* multiple techniques were used to quantitatively analyze the expression of biomarkers in the tumors. *In vivo* multiple technology devices include CCD and charge coupling devices. Figure 6B shows the time-processing image, which decomposes MCF-7 and BT-474 tumors into three time-long paths, indicated by the red, green, and blue monochrome image collections. The expression of biomarkers was determined by integrating the intensity of each component and normalizing the total intensity to synthesize all tumor regions. Figures 6C,D are the results of *in vitro* protein imprinting: Figure 6C shows the result of immunohistochemical experiment of indirect *in vitro* therapy, While Figure 6D shows the result of determined biomarker expression modes of the two tumor hypotypes by using IVM.

At present, this field has become a vital resource for biomedical diagnosis and treatment, and many research groups are competing to continue pushing forward in expanding its study. Several high-level research papers and reviews have been reported recently, such as Guo et al. (2019), who used NIR-II fluorescence and photoacoustic imaging to study the precise identification of the vascular system and micro-tumors in 2019. It is well-known that the diagnosis of cerebrovascular structure with complete blood-brain barrier and microtumors is of great significance for the timely treatment of patients with nervous system diseases. The combined diagnosis and treatment of NIR-II fluorescence and photoacoustic imaging (PAI) is anticipated to provide improving performance, for instance, excellent spatial and temporal distinguishability, large penetration depth and great SNR, and accurate brain diagnosis. In this study, conjugated polymer nanoparticles (CP NPs) with biocompatibility and photostability were prepared, and bimodal brain imaging was achieved using its NIR-II window. The nanoparticles with a uniform size of 50 nm can be prepared by microfluidic device. The emission peak was at 1,156 nm and had strong absorption of 35.2 Lg^−1^cm^−1^ is at 1,000 nm. NIR-II fluorescence imaging provides a solution to the depth and resolution issues previously experienced with the hemodynamic and cerebral vascular systems, providing an imaging depth of 600 microns and a spatial resolution of 23 microns. After an ultrasound-induced opening of the blood-brain barrier, NIR-II PAI could successfully perform non-invasive imaging of deep micro-brain tumors (2.4 mm below dense skull and scalp <2 mm) with a SNR of 7.2. This study showed that CP-NPs is a promising brain diagnostic contrast agent.

In 2019, Fan et al. (2019) published an overview of optical multiplex techniques for biological detection to improve biomedical diagnosis, arguing that traditional methods according to the detection of single disease markers may not be precise enough, since disease progression usually involves a variety of chemicals and biomolecules. Multi-target simultaneous analysis is of great importance in basic biomedical research and clinical application, promoting simultaneous multi-target analysis that requires the development of a high-throughput multi-target biological analysis technology. In order to improve the level of biomedical diagnosis, this paper reviewed the research progress of optical multiplexing analysis technology used in biomedical diagnosis over the recent years. The review primarily focused on the Fluorescence and Surface Enhanced Raman Scattering (SERS) technique which has unique optical characteristics as a main signal reader. The paper also focused on the review of the multiplexing strategy in biomedical field and the recent advances in biosensors from multi-analyte and multi-color cell tracking to multi-channel bioimaging *in vivo*. Finally, the paper provided forecasting of future challenges and opportunities of multi-bio-analysis.

In 2018, He et al. (2018) proposed that the structural design and synthesis of fluorophores emitted in NIR-II biological window are moving toward multi-mode imaging, as well as toward multimodal diagnosis and treatment. He et al. reviewed some progress of NIR-II fluorophores and molecular probes and considered the synthesis of NIR-II fluorescent group fit for multimode imaging will become a new way to obtain high-resolution images through the structural design. NIR-II fluorophores can convert NIR-II photons into heat required for photothermal therapy and can also be stimulated by NIR-II light to generate singlet oxygen for photodynamic therapy. The single probe has both diagnostic and therapeutic functions which can be used for precise treatment. He et al. details the latest development in structural design and synthesis of various NIR-II fluorophores and provides a discussion on the similarities and differences in known NIR-II imaging systems and the recent research on NIR-II imaging in biomedical applications.

### Tracking the Transplanted Stem Cells

Stem cell (SC) is one type of pluripotent cells with self-replication ability, which can differentiate into a variety of functional cells under certain conditions. SC therapy is showing the hope of curing major human diseases. In the process of SC therapy, how to observe the transplanted SCs non-invasively in real time has become a challenge in experimental and clinical research. NIR imaging based on exogenous markers has always been one of the best methods to trace stem cells *in vivo*.

**Figure 7:**
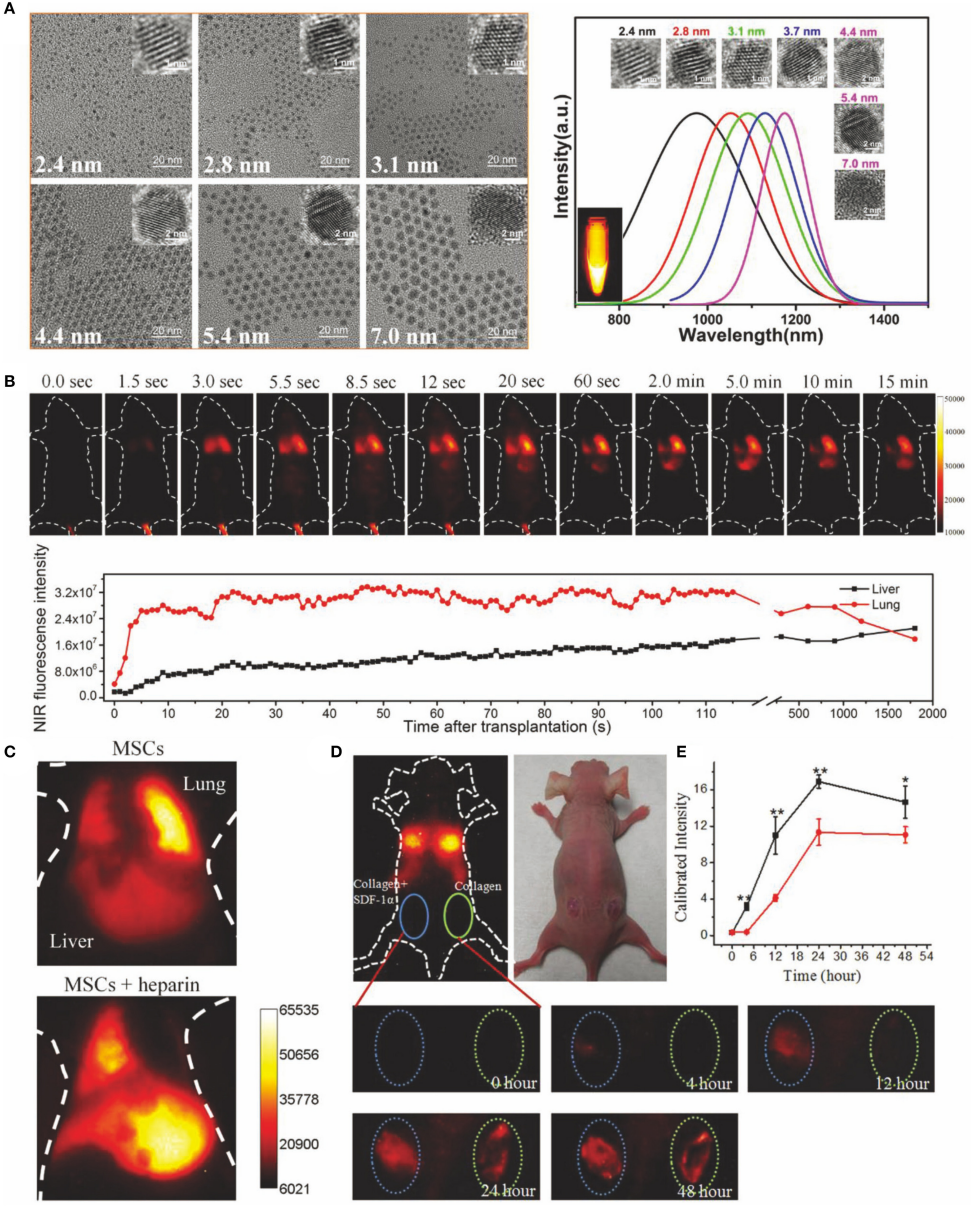
Ag_2_S quantum dots of NIR-II emitting were used to track the transplanted MSCs (Chen et al., 2018b). **(A)** Representative TEM pictures and fluorescence emission spectra of Ag_2_S QDs with diverse sizes. Adapted from Zhang et al. (2014) written by Qiangbin Wang etc. with permission. **(B)** Ag_2_S QDs labeled MSCs were injected into mice by vein, and NIR-II fluorescence imaging was accomplished on the mice with 100 ms exposure. When excited at 808 nm, the InGaAs/SWIR camera was used to obtain the NIR-II image. NIR-II fluorescence signal value in liver and lung of mice at diverse time points was quantitatively analyzed. Adapted from Chen et al. (2018a) written by Qiangbin Wang etc. with permission. **(C)** MSCs were tracked in mice with acute liver failure and labeled by Ag_2_S quantum dots. MSCs were injected into mice in combination with or without heparin and imaged. Adapted from Chen et al. (2013) written by Qiangbin Wang etc. with permission. Homing of MSCs was studied by **(D)** *in vivo* imaging and **(E)** fluorescence quantification. The MSCs were transplanted intravenously into a mouse model with skin trauma. The left trauma was cured with a collagen scaffold loaded with SDF1-α. The right trauma was cured with collagen scaffold. The data are shown as mean ± SD values from *n* = 3, *p < 0.05, **p < 0.01. Adapted from Chen et al. (2015) written by Qiangbin Wang etc. with permission.

In recent years, NIR fluorescent probes, with their strong biological penetration ability, low photon detection domain value, high detection sensitivity, relatively simple operation, and advantages of real-time imaging during surgery, have made the shift of emission from NIR-I window to NIR-II window to track transplanted stem cells. For instance, Chen et al. (2018b) has reviewed the research progress of tracking transplanted SCs with NIR fluorescence NPs. The latest development of NIR-II window fluorescence imaging technology was introduced. The emergence of new fluorescent functional NPs (QDs, RE-doped NPs, organic fluorescent NPs, etc.) makes stem cells easy to be labeled and tracked, greatly promoting the further development of stem cell therapy. NIR-II fluorescence emission is absorbed little by tissues, and the scattering and spontaneous fluorescence in NIR-II region are the least in tissues. Therefore, the signal-to-background ratio can be greatly improved, yielding to a large tissue penetration depths and a good spatial-temporal resolution. Moreover, NIR-II fluorescence imaging technology for accurate optical tracking can improve our comprehending of the fate and regeneration ability of transplanted SCs and provide immense potential development of SC-based regenerative medicine. Up to now, only a few research labs are exploring the feasibility of using NPs emitted in NIR-II to track SCs *in vivo*. Ag_2_S QDs-based NIR-II imaging is currently the only method that has succeeded in SC tracking research *in vivo*. In order to greater improve the performance of NPs emitted in NIR-II in SC-based regeneration research, more efforts should be made to conquer the defects (such as water solubility, physiological stability, biocompatibility, and metabolic capacity) of current NIR-II NP-based imaging technology. In addition, new strategies are needed to take full advantage of NIR-II excitation and emission. The comparison diagram of transplanted mesenchymal stem cells (MSCs) tracked by Ag_2_S quantum dots emitted NIR-II fluorescence is shown in Figure 7.

Figure 7A shows typical TEM pictures and fluorescence emission spectrum of Ag_2_S quantum dots with different particle sizes. Figure 7B shows the real-time NIR-II fluorescence imaging of Ag_2_S QDs labeled MSCs after intravenous injection into mice exposed for 100 ms. When excited at 808 nm, NIR-II fluorescence images were collected with InGaAs/SWIR camera. The fluorescence intensity of NIR-II in liver and lung of mice at diverse time points was measured, and then quantitative analysis was performed. In Figure 7C, Ag_2_S quantum dots were labeled on MSCs and then injected into mice with acute liver failure for tracking study. MSCs were combined with or without heparin and then transplanted intravenously into mice and imaged. Figures 7D,E, respectively show *in vivo* imaging and fluorescence quantitative homing behavior of MSCs to the wound in a mouse model with skin trauma. MSCs were transplanted intravenously into model mice. The left and right wounds were treated with SDF1-α-loaded collagen scaffolds and collagen scaffolds, respectively.

Other related researches on the tracking of transplanted CS with NIR-II quantum dots include an article published in 2019 by Chen's team about the fate of intraarticular injection of MSCs *in vivo* for the useful treatment of supraspinatus tendon tears (Yang et al., 2019). This paper reports that MSCs have a strong therapeutic potential in the treatment of supraspinatus tendon tears. However, by reason of the finite evidence of dynamic visualization of cell behavior *in vivo*, MSC therapy has not been fully utilized and may even be underestimated. Here, PbS QD labeled MSCs can treat supraspinatus tendon tears in mice. PbS QD is a biocompatible NIR-II fluorescence imaging probe, which can provide a cell migration map and information about the biological distribution and clearance process of MSCs injected into the joints with three densities. Intra-articular injection can avoid MSCs being wrapped by filtered organs and reduces organ toxicity induced by quantum dots. It is worth noting that MSCs have similar migration directions, but the migration efficiency of the medium density group is higher. In the repair stage, MSCs stay around the footprint for the longest time, with the highest cell retention rate. In addition, quantitative kinetics studies showed that labeled MSCs were cleared by feces and urine. Histomorphological analysis showed that therapeutic effect of the medium density group was the best, and the labeled MSCs showed no damage or inflammatory response to the main organs, suggesting that too much or too little MSCs administration might reduce its efficacy. This imaging method provides spatio-temporal evidence for the response of MSCs therapy *in vivo*, which is helpful for the optimization of MSCs therapy.

Moreover, in 2018, Huang et al. published a paper on NIR-II fluorescence and bioluminescence multiple imaging for *in vivo* observation of the location, survival and differentiation of transplanted stem cells (Huang et al., 2019). An NIR-II fluorescence/bioluminescence composite imaging method is successfully developed covering 400–1,700nm visible light and NIR-II window for *in vivo* monitoring of location, survival, and osteogenic differentiation of human bone marrow mesenchymal stem cells (hMSCs) in a mouse model of skull defect. The long-term biological distribution of transplanted hMSCs was observed by using Ag_2_S quantum dot with NIR-II window. Bioluminescent imaging (BLI) based on endogenous red firefly luciferase (rfluc) and gaussia luciferase (gluc) driven by collagen type 1 promoter were used to report the survival and osteogenic differentiation status of transplanted hMSCs. At the same time, by combining the three imaging channels, they can not only directly observe the various dynamic biological behaviors of the transplanted hMSCs, but also further observe the promoting effects of immunosuppression and bone morphogenetic protein 2 on the survival and osteogenic differentiation of the transplanted hMSCs. This new multifunctional imaging technique can widely extend the analysis of the fate and therapeutic capacity of transplanted stem cells and help to promote stem cell-based regenerative therapy and its transformation in the clinical applications.

## Conclusion

In summary, compared with traditional NIR-I imaging technology and other medical imaging methods, NIR-II bioimaging technology not only has a deeper imaging depth, but also can better avoid background interference such as spontaneous fluorescence and photon scattering of tissue. So far, a variety of NIR-II dyes, such as inorganic nanomaterials (single-walled carbon nanotubes, semiconductor quantum dots, rare earth nanomaterials), conjugated polymers and organic small-molecule materials, have been successfully synthesized and prepared. Because of its special properties, NIR-II dyes can be used not only as biomedical contrast agent, but also in the fields of photothermal and photodynamic therapy, drug delivery, surgical guidance and tracking the transplanted stem cells. In this article, the reported NIR-II fluorescent probes and their biomedical applications are summarized in Table 1.

**Table 1:** NIR-II contrast agent and its biomedical application.

**Fluorophore**	**Fluorescence property**	**Biomedical applications**	**References**
SWCNTs	808/950–1,400 nm (excitation/emission)	High-resolution imaging of blood vessels	Welsher et al., 2009
M13-SWCNTs	808/950–1,100 nm (excitation/emission)	Targeted fluorescence imaging of tumor tissues in mice	Ghosh et al., 2014
IRDye-800- SWCNTs	808/1,300–1,400 nm (excitation/emission)	Imaging of the distribution of veins in the head of mice and the fine structure of the brain capillaries	Hong et al., 2014
A synthetic organic molecule (CH1055)	750/1,055 nm (excitation/emission)	Tumor imaging and precise image-guided tumor-removal surgery	Antaris et al., 2016
o-SWCNT-PEG	980/1,300 nm (excitation/emission)	Used in angiographic imaging	Takeuchi et al., 2019
Ag_2_S QD	800/1,200 nm (excitation/emission)	Used in NIR-II cell imaging and non-specific tumor detection	Hong et al., 2012b; Zhang et al., 2012, 2013
Au/Cu alloy nanodots or Au/Co alloy nanodots	808/950–1,100 nm (excitation/emission)	It is a non-toxic and multifunctional nanodots for used in biological imaging, especially in multi-mode imaging	Andolina et al., 2013; Marbella et al., 2014
NaYF_4_:Yb/Ln@NaYF_4_(Ln= Er, Ho, Tm, Pr)nanocrystals	800/~1,500 nm (excitation/emission)	Widely applied *in vivo* bioimaging research	Naczynski et al., 2013
SrF_2_:Nd^3+^ NP	808/1,100 nm (excitation/emission)	*In vivo* NIR-II optical imaging	Villa et al., 2015
NaYF_4_:Yb^3+^/X^3+^@NaYbF_4_@NaYF_4_: Nd^3+^ (X = null, Er, Ho, Tm or Pr)	700–860/1,000–1,600 nm (excitation/emission)	NIR-II *in vivo* imaging of deep tissue	Shao et al., 2016
FD-1080	1,046/1,080 nm (excitation/emission)	non-intrusive high distinguishability deep tissue hindlimb vascular system and brain veins biological imaging	Li et al., 2018
Free ICG	810–830/1,100 nm (excitation/emission)	Utility in NIR-II navigated cytoreductive surgery of cerebral cancers and accelerate the clinical translation of NIR-II imaging technology	Starosolski et al., 2017; Zeh et al., 2017; Cho et al., 2019a,b; Wang et al., 2019b
liposomal-ICG (Lip-ICG)	782/950–1500 nm (excitation/emission)	Long-term imaging of hind limb and intracranial vessels *in vivo*	Bhavane et al., 2018
ICG conjugated bevacizumab (Bev-ICG)	808 nm/>900 nm (excitation/emission)	Immensely facilitate the application of NIR-II clinical bioimaging	Suo et al., 2019
Core:DCNPs(NaGdF4:5%Nd@NaGdF4) *In vivo* assembly nanoprobe: DCNPs-L_1_/L_2_-FSH_β_(L1 and L2 are conjugated DNA; FSH_β_ is a follicle stimulating hormone peptide)	808/1,060 nm (excitation/emission)	Tumor imaging and image-guided surgery	Wang et al., 2018
Ag_2_Te quantum dots (QDs)	808/1,300 nm (excitation/emission)	Tumor imaging and image-guided surgery	Zhang et al., 2019
lanthanide complex (Nd-DOTA)	808/1,060 and 1,330 nm (excitation/emission)	Accurately mark the profile of micro-tumors in surgery and thoroughly removed under the guidance of NIR-II biological imaging	Yang et al., 2018
semiconductor polymer nanoparticle of diketopyrrolopyrrole (PDFT1032)	809/1,032 nm (excitation/emission)	Widely used in clinical imaging and surgical treatment of malignant tumors	Shou et al., 2018
NaErF_4_:2%Ho@NaYF_4_ UCNPs the sensor is based on the ratio meter fluorescence	1,530/1,180 nm (excitation/emission)	Used to quantify the degree of actual inflammation in clinical tests	Liu et al., 2018
DCNPs(NaGdF_4_:5%Nd@NaGdF_4_)@GSH	808/1,060 nm (excitation/emission)	Accurate imaging of inflammation *in vivo*	Zhao et al., 2019
pentamethine cyanine NIR-II molecular fluorophore	1,015/1,065 nm (excitation/emission)	high-quality imaging and pH sensing *in vivo*	Wang et al., 2019a
Peroxynitrate NIR-II molecular fluorophore IRBTP-O	575 and 905/>1,000 nm (excitation/emission)	Drug-induced hepatotoxicity detection	Li et al., 2019c
Polyacrylic acid (PAA) modified NaLuF_4_:Gd/Nd nanorods (PAA-NRs)	808/1,056 and 1,328 nm (excitation/emission)	For Fluorescence imaging-guided detection of small tumors, angiogenesis-related diseases, and angiogenesis diagnosis	Li et al., 2019a
NaLuF_4_: Gd/Yb/Er NRs@PDA	980/1,525 nm (excitation/emission)	Visualization of tumor-related vascular malformations and imaging-guided photothermal therapy	Li et al., 2019b
A bright organic NIR-II fluorescent nanocluster (p-FE)	980/1,100 nm (excitation/emission)	As a vascular imaging reagent for non-invasive *in vivo* blood flow tracking in the rat cerebrovascular system	Wan et al., 2018
NaGdF_4_@NaGdF_4_:Yb,Er@NaYF_4_: Yb@NaNdF_4_:Yb	808 nm/Er^3+^ at 1,525 nm. Ho^3+^ at 1,155 nm, Pr^3+^ at 1,289 nm, Tm^3+^ at 1,475 nm Nd^3+^ at 1,060 nm (excitation/emission)	As a microinvasive probes for disease diagnosis	Fan et al., 2018
Conjugated polymer nanoparticles (CP NPs)	980/1,156 nm (excitation/emission)	Accurate brain diagnosis	Guo et al., 2019
Ag_2_S QDs	808/From 900 to 1,700 nm (excitation/emission)	Tracking the transplanted stem cells	Chen et al., 2013, 2015, 2018a; Zhang et al., 2014; Huang et al., 2019
C_18_-PMH-PEG-Ag_2_Se	808/1,300 nm (excitation/emission)	Tracking the transplanted stem cells	Dong et al., 2013; Chen et al., 2018b
PbS QDs	808/1,100 nm (excitation/emission)	Tracking the transplanted stem cells *in vivo* to provide evidence for therapy and promote the optimization of therapy	Yang et al., 2019
AgInTe_2_ QDs	700 nm/between 1,095 and 1,160 nm (excitation/emission)	*In vivo* bioimaging and solar energy conversion systems	Langevin et al., 2015; Kameyama et al., 2016; Chen et al., 2018b

With the development of molecular design theory and nanomaterials, more and more NIR-II imaging systems will be designed, developed and promoted into clinical trials. It is worth noting that at present, most of NIR-II fluorescent molecules have poor water solubility and physiological stability, and low fluorescence quantum yield. For polymers and inorganic nano systems, problems, such as slow metabolism, high toxicity, and lack of specific tissue targeting, still exist. New methods and materials will be helpful to promote the application of NIR-II imaging technology in the field of biology. In general, the following points should be paid attention to in the development and design of NIR-II dyes. Firstly, in order to improve the water solubility and physiological stability of organic NIR-II fluorescent dyes, reduce the band gap when choosing the appropriate space configuration, and consider the influence of the group on the water solubility of dyes when modifying the group. Secondly, in order to improve the biocompatibility and metabolic capacity of the probe, the molecular structure should be designed reasonably. In particular, when the probe is a polymer molecule dye and nanoparticles, the design and synthesis of the degradation of NIR-II dye probe is the best. Thirdly, dye is endowed with specific functions, such as combining dye with targeted molecules, improving the aggregation ability of dye in specific parts of the organism, and promoting the development of disease targeting and early diagnosis in clinical practice.

## Author Contributions

JC wrote the draft and corresponded to submittal, revision, and coordination. BZ, KZ, SH, LM, JS, and HY participated in manuscript revision. JS and HY oversaw the design, quality assurance, and finalization of the manuscript.

### Conflict of Interest

The authors declare that the research was conducted in the absence of any commercial or financial relationships that could be construed as a potential conflict of interest.

## References

[B1] Andolina C. M. Dewar A. C. Smith A. M. Marbella L. E. Hartmann M. J. Millstone J. E. (2013). Photoluminescent gold-copper nanoparticle alloys with composition-tunable near-infrared emission. J. Am. Chem. Soc. 135, 5266–5269. 10.1021/ja400569u 23548041

[B2] Antaris A. L. Chen H. Cheng K. Sun Y. Hong G. S. Qu C. R.. (2016). A small-molecule dye for NIR-II imaging. Nat. Mater. 15, 235–242. 10.1038/nmat4476 26595119

[B3] Bashkatov A. N. Genina E. A. Kochubey V. I. Tuchin V. V. (2005). Optical properties of human skin, subcutaneous and mucous tissues in the wavelength range from 400 to 2000 nm. J. Phys. D Appl. Phys. 38:2543–2555. 10.1088/0022-3727/38/15/004

[B4] Bhavane R. Starosolski Z. Stupin I. Ghaghada K. B. Annapragada A. (2018). NIR-II fluorescence imaging using indocyanine green nanoparticles. Sci. Rep. 8:14455. 10.1038/s41598-018-32754-y 30262808 PMC6160486

[B5] Chan E. K. Sorg B. Protsenko D. O'Neil M. Motamedi M. Welch A. J. (1996). Effects of compression on soft tissue optical properties. IEEE J. Sel. Top. Quantum Electron. 2, 943–950. 10.1109/2944.577320

[B6] Chen G. C. Lin S. Y. Huang D. H. Zhang Y. J. Li C. Y. Wang M.. (2018a). Revealing the fate of transplanted stem cells *in vivo* with a novel optical imaging strategy. Small 14:1702679. 10.1002/smll.201702679 29171718

[B7] Chen G. C. Tian F. Li C. Y. Zhang Y. J. Weng Z. Zhang Y.. (2015). *In vivo* real-time visualization of mesenchymal stem cells tropism for cutaneous regeneration using NIR-II fluorescence imaging. Biomaterials 53, 265–273. 10.1016/j.biomaterials.2015.02.090 25890725

[B8] Chen G. C. Tian F. Zhang Y. Zhang Y. J. Li C. Y. Wang Q. B. (2013). Tracking of transplanted human mesenchymal stem cells in living mice using near-infrared Ag_2_S quantum dots. Adv. Funct. Mater. 24, 2481–2488. 10.1002/adfm.201303263

[B9] Chen G. C. Zhang Y. J. Li C. Y. Huang D. H. Wang Q. W. Wang Q. B. (2018b). Recent advances in tracking the transplanted stem cells using near-infrared fluorescent nanoprobes: turning from the first to the second near-infrared window. Adv. Funct. Mater. 7, 1–18. 10.1002/adhm.201800497 30019509

[B10] Chen G. Y. Ohulchanskyy T. Y. Liu S. Law W. C. Wu F. Swihart M. T. (2012). Core/shell NaGdF_4_: Nd^3+^/NaGdF_4_ nanocrystals with efficient near-infrared to near-infrared downconversion photoluminescence for bioimaging applications. ACS Nano. 6, 2969–2977. 10.1021/nn2042362 22401578 PMC3430515

[B11] Chen G. Y. Qiu H. L. Prasad P. N. Chen X. Y. (2014). Upconversion nanoparticles: design, nanochemistry, and applications in theranostics. Chem. Rev. 114, 5161–5214. 10.1021/cr400425h 24605868 PMC4039352

[B12] Cho S. S. Salinas R. De Ravin E. Teng C. W. Li C. Abdullah K. G. (2019a). Near-infrared imaging with second-window indocyanine green in newly diagnosed high-grade gliomas predicts gadolinium enhancement on postoperative magnetic resonance imaging. Mol. Imaging Biol. 21, 1–11. 10.1007/s11307-019-01455-x PMC8613832 31712948

[B13] Cho S. S. Salinas R. Lee J. Y. K. (2019b). Indocyanine-green for fluorescence-guided surgery of brain tumors: evidence, techniques, and practical experience. Front. Surg. 6:11. 10.3389/fsurg.2019.00011 30915339 PMC6422908

[B14] Cosco E. D. Caram J. R. Bruns O. T. Franke D. Day R. A. Farr E. P.. (2017). Flavylium polymethine fluorophores for near-and shortwave infrared imaging. Angew. Chem. Int. Ed. Engl. 56, 13126–13129. 10.1002/anie.201706974 28806473

[B15] Croce A. C. Bottiroli G. (2014). Autofluorescence spectroscopy and imaging: a tool for biomedical research and diagnosis. Eur. J. Histochem. 58:2461. 10.4081/ejh.2014.2461 25578980 PMC4289852

[B16] Diao S. Blackburn J. L. Hong G. S. Antaris A. L. Chang J. L. Wu J. Z.. (2015). Fluorescence imaging *in vivo* at wavelengths beyond 1500 nm. Angew. Chem. Int. Ed. Engl. 54, 14758–14762. 10.1002/anie.201507473 26460151

[B17] Dong B. H. Li C. Y. Chen G. C. Zhang Y. J. Zhang Y. Deng M. J. (2013). Facile synthesis of highly photoluminescent Ag_2_Se quantum dots as a new fluorescent probe in the second near-infrared window for *in vivo* imaging. Chem. Mater. 25, 2503–2509. 10.1021/cm400812v

[B18] Du Y. Hu X. H. Cariveau M. Ma X. Kalmus G. W. Lu J. Q. (2001). Optical properties of porcine skin dermis between 900 nm and 1500 nm. Phys. Med. Biol. 46, 167–181. 10.1088/0031-9155/46/1/312 11197670

[B19] Fan Y. Wang P. Y. Lu Y. Q. Wang R. Zhou L. Zheng X. L.. (2018). Lifetime-engineered NIR-II nanoparticles unlock multiplexed *in vivo* imaging. Nat. Nanotechnol. 13, 941–946. 10.1038/s41565-018-0221-0 30082923

[B20] Fan Y. Wang S. F. Zhang F. (2019). Optical multiplexed bioassays for improve biomedical diagnostics. Angew. Chem. Int. Ed. Engl. 58, 13208–13219. 10.1002/anie.201901964 30994960

[B21] Ferrando R. Jellinek J. Johnston R. L. (2008). Nanoalloys: from theory to applications of alloy clusters and nanoparticles. Chem. Rev. 108, 845–910. 10.1021/cr040090g 18335972

[B22] Frangioni J. V. (2003). *In vivo* near-infrared fluorescence imaging. Curr. Opin. Chem. Biol. 7, 626–634. 10.1016/j.cbpa.2003.08.007 14580568

[B23] Friebel M. Helfmann J. Netz U. Meinke M. (2009). Influence of oxygen saturation on the optical scattering properties of human red blood cells in the spectral range 250 to 2000 nm. J. Biomed. Opt. 14:034001. 10.1117/1.3127200 19566295

[B24] Ghosh D. Bagley A. F. Na Y. J. Birrer M. J. Bhatia S. N. Belcher A. M. (2014). Deep, noninvasive imaging and surgical guidance of submillimeter tumors using targeted M13-stabilized single-walled carbon nanotubes. Proc. Natl. Acad. Sci. U.S.A. 111, 13948–13953. 10.1073/pnas.1400821111 25214538 PMC4183329

[B25] Gui R. J. Sun J. Liu D. X. Wang Y. F. Jin H. (2014a). A facile cation exchange-based aqueous synthesis of highly stable and biocompatible Ag_2_S quantum dots emitting in the second near-infrared biological window. Dalton Trans. 43, 16690–16697. 10.1039/C4DT00699B 25270003

[B26] Gui R. J. Wan A. J. Liu X. F. Yuan W. Jin H. (2014b). Water-soluble multidentate polymers compactly coating Ag_2_S quantum dots with minimized hydrodynamic size and bright emission tunable from red to second near-infrared region. Nanoscale 6, 5467–5473. 10.1039/c4nr00282b 24728046

[B27] Guo B. Feng Z. Hu D. H. Xu S. D. Middha E. Pan Y. T.. (2019). Precise deciphering of brain vasculatures and microscopic tumors with dual NIR-II fluorescence and photoacoustic imaging. Adv. Mater. 31:e1902504. 10.1002/adma.201902504 31169334

[B28] He S. Q. Song J. Qu J. L. Cheng Z. (2018). Crucial breakthrough of second near-infrared biological window fluorophores: design and synthesis toward multimodal imaging and theranostics. Chem. Soc. Rev. 47, 4258–4278. 10.1039/C8CS00234G 29725670

[B29] Hemmer E. Benayas A. Légaré F. Vetrone F. (2016). Exploiting the biological windows: current perspectives on fluorescent bioprobes emitting above 1000 nm. Nanoscale Horiz. 1, 168–184. 10.1039/C5NH00073D 32260620

[B30] Hilderbrand S. A. Weissleder R. (2010). Near-infrared fluorescence: application to *in vivo* molecular imaging, Curr. Opin. Chem. Biol. 14, 71–79. 10.1016/j.cbpa.2009.09.029 19879798

[B31] Hong G. S. Diao S. Chang J. L. Antaris A. L. Chen C. X. Zhang B.. (2014). Through skull fluorescence imaging of the brain in a new near-infrared window. Nat. Photonics. 8, 723–730. 10.1038/nphoton.2014.166 27642366 PMC5026222

[B32] Hong G. S. Lee J. C. Robinson J. T. Raaz U. Xie L. M. Huang N. F.. (2012a). Multifunctional *in vivo* vascular imaging using near-infrared II fluorescence. Nat. Med. 18, 1841–1846. 10.1038/nm.2995 23160236 PMC3595196

[B33] Hong G. S. Robinson J. T. Zhang Y. J. Diao S. Antaris A. L. Wang Q. B.. (2012b). *In vivo* fluorescence imaging with Ag_2_S quantum dots in the second near-infrared region. Angew. Chem. Int. Ed. Engl. 51, 9818–9821. 10.1002/anie.201206059 22951900

[B34] Huang D. H. Lin S. Y. Wang Q. W. Zhang Y. J. Li C. Y. Ji R. (2019). An NIR-II fluorescence/dual bioluminescence multiplexed imaging for *in vivo* visualizing the location, survival, and differentiation of transplanted stem cells. Adv. Funct. Mater. 29, 1–11. 10.1002/adfm.201806546

[B35] Jiang X. Y. Cao C. Feng W. Li F. Y. (2016). Nd^3+^-doped LiYF_4_ nanocrystals for bio-imaging in the second near-infrared window. J. Mater. Chem. B 4, 87–95. 10.1039/C5TB02023A 32262811

[B36] Kameyama T. Ishigami Y. Yukawa H. Shimada T. Baba Y. Ishikawa T.. (2016). Crystal phase-controlled synthesis of rod-shaped AgInTe_2_ nanocrystals for *in vivo* imaging in the near-infrared wavelength region. Nanoscale 8, 5435–5440. 10.1039/C5NR07532G 26899620

[B37] Kobayashi H. Ogawa M. Alford R. Choyke P. L. Urano Y. (2010). New strategies for fluorescent probe design in medical diagnostic imaging. Chem. Rev. 110, 2620–2640. 10.1021/cr900263j 20000749 PMC3241938

[B38] Langevin M. A. Pons T. Ritcey A. M. Allen C. N. (2015). Near-infrared emitting AgInTe_2_ and Zn-Ag-In-Te colloidal nanocrystals. Nanoscale Res. Lett. 10:255. 10.1186/s11671-015-0951-y 26058512 PMC4477004

[B39] Lavis L. D. Raines R. T. (2008). Bright ideas for chemical biology. ACS Chem. Biol. 3, 142–155. 10.1021/cb700248m 18355003 PMC2802578

[B40] Li B. H. Lu L. F. Zhao M. Y. Lei Z. H. Zhang F. (2018). Efficient 1064-nm NIR-II excitation fluorescent molecular dye for deep-tissue high-resolution dynamic bioimaging. Angew. Chem. Int. Ed. Engl. 57, 7483–7487. 10.1002/anie.201801226 29493057

[B41] Li D. D. Wang S. F. Lei Z. H. Sun C. X. El-Toni A. M. Alhoshan M. S.. (2019c). Peroxynitrite activatable NIR-II fluorescent molecular probe for drug-induced hepatotoxicity monitoring. Anal. Chem. 91, 4771–4779. 10.1021/acs.analchem.9b00317 30808169

[B42] Li X. L. Jiang M. Y. Li Y. B. Xue Z. L. Zeng S. J. Liu H. R. (2019a). 808 nm laser-triggered NIR-II emissive rare-earth nanoprobes for small tumor detection and blood vessel imaging. Mater. Sci. Eng. C Mater. Biol. Appl. 100, 260–268. 10.1016/j.msec.2019.02.106 30948060

[B43] Li X. L. Jiang M. Y. Zeng S. J. Liu H. R. (2019b). Polydopamine coated multifunctional lanthanide theranostic agent for vascular malformation and tumor vessel imaging beyond 1500 nm and imaging-guided photothermal therapy. Theranostics 9, 3866–3878. 10.7150/thno.31864 31281519 PMC6587345

[B44] Li X. M. Wang R. Zhang F. Zhou L. Shen D. K. Yao C.. (2013). Nd^3+^ sensitized up/down converting dual-mode nanomaterials for efficient *in-vitro* and *in-vivo* bioimaging excited at 800 nm. Sci. Rep. 3:3536. 10.1038/srep03536 24346622 PMC3866591

[B45] Lim Y. T. Kim S. Nakayama A. Stott N. E. Bawendi M. G. Frangioni J. V. (2003) Selection of quantum dot wavelengths for biomedical assays imaging. Mol. Imaging 2, 50–64. 10.1162/153535003765276282 12926237

[B46] Liu L. Wang S. F. Zhao B. Z. Pei P. Fan Y. Li X. M.. (2018). Er^3+^ sensitized 1530 nm to 1180 nm second near-infrared window upconversion nanocrystals for *in vivo* biosensing. Angew. Chem. Int. Ed. Engl. 57, 7518–7522. 10.1002/anie.201802889 29719100

[B47] Lu H. Mack J. Yang Y. C. Shen Z. (2014). Structural modification strategies for the rational design of red/NIR region BODIPYs. Chem. Soc. Rev. 43, 4778–4823. 10.1039/C4CS00030G 24733589

[B48] Ma Q. Su X. G. (2010). Near-infrared quantum dots: synthesis, functionalization and analytical applications. Analyst 135, 1867–1877. 10.1039/c0an00233j 20563343

[B49] Marbella L. E. Andolina C. M. Smith A. M. Hartmann M. J. Dewar A. C. Johnston K. A. (2014). Gold-cobalt nanoparticle alloys exhibiting tunable compositions, near-infrared emission, and high T-2 relaxivity. Adv. Funct. Mater. 24, 6532–6539. 10.1002/adfm.201400988

[B50] Mishra A. Behera R. K. Behera P. K. Mishra B. K. Behera G. B. (2000). Cyanines during the 1990s: A Review. Chem. Rev. 100, 1973–2011. 10.1021/cr990402t 11749281

[B51] Murakami T. Nakatsuji H. Inada M. Matoba Y. Umeyama T. Tsujimoto M.. (2012). Photodynamic and photothermal effects of semiconducting and metallic-enriched single-walled carbon nanotubes. J. Am. Chem. Soc. 134, 17862–17865. 10.1021/ja3079972 23083004

[B52] Naczynski D. J. Tan M. C. Riman R. E. Moghe P. V. (2014) Rare earth nanoprobes for functional biomolecular imaging theranostics. J. Mater. Chem. B 2, 2958–2973. 10.1039/C4TB00094C 24921049 PMC4048749

[B53] Naczynski D. J. Tan M. C. Zevon M. Wall B. Kohl J. Kulesa A.. (2013). Rare-earth-doped biological composites as *in vivo* shortwave infrared reporters. Nat. Commun. 4:2199. 10.1038/ncomms3199 23873342 PMC3736359

[B54] O'Connell M. J. Bachilo S. M. Huffman C. B. Moore V. C. Strano M. S. Haroz E. H.. (2002). Band gap fluorescence from individual single-walled carbon nanotubes. Science 297, 593–596. 10.1126/science.1072631 12142535

[B55] Peters V. G. Wyman D. R. Patterson M. S. Frank G. L. (1990). Optical properties of normal and diseased human breast tissues in the visible and near infrared. Phys. Med. Biol. 35, 1317–1334. 10.1088/0031-9155/35/9/010 2236211

[B56] Pokhrela M. Mimun L. C. Yust B. Kumar G. A. Dhanale A. Tang L. (2014). Stokes emission in GdF_3_:Nd^3+^ nanoparticles for bioimaging probes. Nanoscale 6, 1667–1674. 10.1039/C3NR03317A 24336743 PMC4274780

[B57] Prahl S. A. van Gemert M. J. C. Welch A. J. (1993). Determining the optical properties of turbid media by using the adding-doubling method. Appl. Opt. 32, 559–568. 10.1364/AO.32.000559 20802725

[B58] Robinson J. T. Welsher K. Tabakman S. M. Sherlock S. P. Wang H. L. Luong R.. (2010). High performance *in vivo* near-IR (> 1 μm) imaging and photothermal cancer therapy with carbon nanotubes. Nano Res. 3, 779–793. 10.1007/s12274-010-0045-1 21804931 PMC3143483

[B59] Schnermann M. J. (2017). Organic dyes for deep bioimaging. Nature 551, 176–177. 10.1038/nature24755 29088708

[B60] Sevick-Muraca E. M. Houston J. P. Gurfinkel M. (2002). Fluorescence-enhanced, near infrared diagnostic imaging with contrast agents. Curr. Opin. Chem. Biol. 6, 642–650. 10.1016/S1367-5931(02)00356-3 12413549

[B61] Shao W. Chen G. Y. Kuzmin A. Kutscher H. L. Pliss A. Ohulchanskyy T. Y.. (2016). Tunable narrow band emissions from dye-sensitized core/shell/shell nanocrystals in the second near-infrared biological window. J. Am. Chem. Soc. 138, 16192–16195. 10.1021/jacs.6b08973 27935695 PMC5474680

[B62] Shou K. Q. Tang Y. F. Chen H. Chen S. Zhang L. Zhang A.. (2018). Diketopyrrolopyrrole-based semiconducting polymer nanoparticles for *in vivo* second nearinfrared window imaging and image-guided tumor surgery. Chem. Sci. 9, 3105–3110. 10.1039/C8SC00206A 29732093 PMC5914543

[B63] Simpson C. R. Kohl M. Essenpreis M. Cope M. (1998). Near-infrared optical properties of *ex vivo* human skin and subcutaneous tissues measured using the Monte Carlo inversion technique. Phys. Med. Biol. 43, 2465–2478. 10.1088/0031-9155/43/9/003 9755939

[B64] Sinkeldam R. W. Greco N. J. Tor Y. (2010). Fluorescent analogs of biomolecular building blocks: design, properties, and applications. Chem. Rev. 110, 2579–2619. 10.1021/cr900301e 20205430 PMC2868948

[B65] Smith A. M. Mancini M. C. Nie S. (2009). Bioimaging: second window for *in vivo* imaging. Nat. Nanotechnol. 4, 710–711. 10.1038/nnano.2009.326 19898521 PMC2862008

[B66] Starosolski Z. Bhavane R. Ghaghada K. B. Vasudevan S. A. Kaay A. Annapragada A. (2017). Indocyanine green fluorescence in second near-infrared (NIR-II) window. PLoS ONE 12, 1–14. 10.1371/journal.pone.0187563 29121078 PMC5679521

[B67] Suo Y. K. Wu F. X. Xu P. F. Shi H. Wang T. Z. Liu H. G.. (2019). NIR-II fluorescence endoscopy for targeted imaging of colorectal cancer. Adv. Healthc. Mater. 8:1900974. 10.1002/adhm.201900974 31697035

[B68] Takeuchi T. Iizumi Y. Yudasaka M. Kizaka-Kondoh S. Okazaki T. (2019). Characterization and biodistribution analysis of oxygen-doped single-walled carbon nanotubes used as *in vivo* fluorescence imaging probes. Bioconjugate Chem. 30, 1323–1330. 10.1021/acs.bioconjchem.9b00088 30848886

[B69] Tan M. C. Kumar G. A. Riman R. E. Brik M. G. Brown E. Hommerich U. (2009). Synthesis and optical properties of infrared-emitting YF3: Nd nanoparticles. J. Appl. Phys. 106:063118 10.1063/1.3168442

[B70] Thekkek N. Richards-Kortum R. (2008). Optical imaging for cervical cancer detection: solutions for a continuing global problem. Nat. Rev. Cancer 8, 725–731. 10.1038/nrc2462 19143057 PMC2633464

[B71] Troy T. L. Thennadil S. N. (2001). Optical properties of human skin in the near infrared wavelength range of 1000 to 2200 nm. J. Biomed. Opt. 6, 167–176. 10.1117/1.1344191 11375726

[B72] van Saders B. Al-Baroudi L. Tan M. C. Riman R. E. (2013). Rare-earth doped particles with tunable infrared emissions for biomedical imaging. Opt. Mater. Express 3, 566–573. 10.1364/OME.3.000566

[B73] Villa I. Vedda A. Cantarelli I. X. Pedroni M. Piccinelli F. Bettinelli M. (2015). 1.3 μm emitting SrF_2_: Nd^3+^ nanoparticles for high contrast *in vivo* imaging in the second biological window. Nano Res. 8, 649–665. 10.1007/s12274-014-0549-1

[B74] Wan H. Yue J. Y. Zhu S. J. Uno T. Zhang X. D. Yang Q. L.. (2018). A bright organic NIR-II nanofluorophore for three-dimensional imaging into biological tissues. Nat. Commun. 9, 1171–1180. 10.1038/s41467-018-03505-4 29563581 PMC5862886

[B75] Wang P. Y. Fan Y. Lu L. F. Liu L. Fan L. L. Zhao M. Y.. (2018). NIR-II nanoprobes in-vivo assembly to improve image-guided surgery for metastatic ovarian cancer. Nat. Commun. 9, 2898–2908. 10.1038/s41467-018-05113-8 30042434 PMC6057964

[B76] Wang P. Y. Wang X. D. Luo Q. Li Y. Lin X. X. Fan L. L.. (2019b). Fabrication of red blood cell-based multimodal theranostic probes for second near-infrared window fluorescence imaging-guided tumor surgery and photodynamic therapy. Theranostics 9, 369–380. 10.7150/thno.29817 30809280 PMC6376196

[B77] Wang S. F. Fan Y. Li D. D. Sun C. X. Lei Z. H. Lu L. F.. (2019a). Anti-quenching NIR-II molecular fluorophores for *in vivo* high-contrast imaging and pH sensing. Nat. Commun. 10, 1–11. 10.1038/s41467-019-09043-x 30837470 PMC6401027

[B78] Weissleder R. (2001). A clearer vision for *in vivo* imaging. Nat. Biotechnol. 19, 316–317. 10.1038/86684 11283581

[B79] Welsher K. Liu Z. Sherlock S. P. Robinson J. T. Chen Z. Daranciang D.. (2009). A route to brightly fluorescent carbon nanotubes for near-infrared imaging in mice. Nat. Nanotechnol. 4, 773–780. 10.1038/nnano.2009.294 19893526 PMC2834239

[B80] Welsher K. Sherlock S. P. Dai H. J. (2011). Deep-tissue anatomical imaging of mice using carbon nanotube fluorophores in the second near-infrared window. Proc. Natl. Acad. Sci. U.S.A. 108, 8943–8948. 10.1073/pnas.1014501108 21576494 PMC3107273

[B81] Willmann J. K. van Bruggen N. Dinkelborg L. M. Gambhir S. S. (2008). Molecular imaging in drug development. Nat. Rev. Drug Discov. 7, 591–607. 10.1038/nrd2290 18591980

[B82] Wu W. Yang Y. Q. Yang Y. Yang Y. M. Zhang K. Y. Guo L.. (2019). Molecular engineering of an organic NIR-II fluorophore with aggregation-induced emission characteristics for *in vivo* imaging. Small 15, 1–10. 10.1002/smll.201970106 30925013

[B83] Yang H. Y. Zhao Y. W. Zhang Z. Y. Xiong H. M. Yu S. N. (2013). One-pot synthesis of water-dispersible Ag_2_S quantum dots with bright fluorescent emission in the second near-infrared window. Nanotechnology. 24:055706. 10.1088/0957-4484/24/5/055706 23324261

[B84] Yang Q. L. Ma Z. R. Wang H. S. Zhou B. Zhu S. J. Zhong Y. T.. (2017). Rational design of molecular fluorophores for biological imaging in the NIR-II window. Adv. Mater. 29:1605497. 10.1002/adma.201605497 28117499

[B85] Yang Y. L. Wang P. Y. Lu L. Y. Fan Y. Sun C. X. Fan L. L.. (2018). Small-molecule lanthanide complexes probe for second near-infrared window bioimaging. Anal. Chem. 90, 7946–7952. 10.1021/acs.analchem.8b00603 29865784

[B86] Yang Y. M. Chen J. Shang X. L. Feng Z. J. Chen C. Lu J. Y.. (2019). Visualizing the fate of intra-articular injected mesenchymal stem cells *in vivo* in the second near-infrared window for the effective treatment of supraspinatus tendon tears. Adv. Sci. 6, 1–12. 10.1002/advs.201901018 31592419 PMC6774022

[B87] Yi H. J. Ghosh D. Ham M. H. Qi J. F. Barone P. W. Strano M. S.. (2012). M13 phage-functionalized single-walled carbon nanotubes as nanoprobes for second near-infrared window fluorescence imaging of targeted tumors. Nano Lett. 12, 1176–1183. 10.1021/nl2031663 22268625 PMC3905603

[B88] Zeh R. Sheikh S. Xia L. Pierce J. Newton A. Predina J.. (2017). The second window ICG technique demonstrates a broad plateau period for near infrared fluorescence tumor contrast in glioblastoma. PLoS ONE 12:e0182034. 10.1371/journal.pone.0182034 28738091 PMC5524327

[B89] Zhang J. J. Lin Y. Zhou H. He H. Ma J. J. Luo M. Y. (2019). Cell membrane-camouflaged NIR II fluorescent Ag_2_Te quantum dots-based nanobioprobes for enhanced *in vivo* homotypic tumor imaging. Adv. Healthc. Mater. 8:e1900341 10.1002/adhm.201900341 31125518

[B90] Zhang Y. Hong G. H. Zhang Y. J. Chen G. C. Li F. Dai H. J.. (2012). Ag_2_S quantum dot: a bright and biocompatible fluorescent nanoprobe in the second near-infrared window. ACS Nano 6, 3695–3702. 10.1021/nn301218z 22515909 PMC3358570

[B91] Zhang Y. Zhang Y. J. Hong G. S. He W. Zhou K. Yang K.. (2013). Biodistribution, pharmacokinetics and toxicology of Ag_2_S near-infrared quantum dots in mice. Biomaterials 34, 3639–3646. 10.1016/j.biomaterials.2013.01.089 23415643

[B92] Zhang Y. J. Liu Y. S. Li C. Y. Chen X. Y. Wang Q. B. (2014). Controlled synthesis of Ag_2_S quantum dots and experimental determination of the exciton bohr radius. J. Phys. Chem. C 118, 4918–4923. 10.1021/jp501266d

[B93] Zhao M. Y. Wang R. Li B. H. Fan Y. Wu Y. F. Zhu X. Y.. (2019). Precise *in vivo* inflammation imaging using *in-situ* responsive cross-linking of glutathione modified ultra-small NIR-II lanthanide nanoparticles. Angew. Chem. Int. Ed. Engl. 58, 2050–2054. 10.1002/anie.201812878 30589175

[B94] Zhao Y. X. Song Z. M. (2014). Phase transfer-based synthesis of highly stable, biocompatible and the second near-infrared-emitting silver sulfide quantum dots. Mater. Lett. 126, 78–80. 10.1016/j.matlet.2014.04.014

[B95] Zheng M. Jagota A. Semke E. D. Diner B. A. Mclean R. S. Lustig S. R.. (2003). DNA-assisted dispersion and separation of carbon nanotubes. Nat. Mater. 2, 338–342. 10.1038/nmat877 12692536

[B96] Zhu S. J. Tian R. Antaris A. L. Chen X. Y. Dai H. J. (2019). Near-infrared-II molecular dyes for cancer imaging and surgery. Adv. Mater. 31:1900321. 10.1002/adma.201900321 31025403 PMC6555689

[B97] Zhu S. J. Yung B. C. Chandra S. Niu G. Antaris A. L. Chen X. Y. (2018). Near-infrared-II (NIR-II) bioimaging via off-peak NIR-I fluorescence emission. Theranostics 8,4141–4151. 10.7150/thno.27995 30128042 PMC6096392

